# Relativity as a Synthesis Design Principle: A Comparative Study of [3 + 2] Cycloaddition of Technetium(VII) and Rhenium(VII) Trioxo Complexes with Olefins

**DOI:** 10.1021/acs.inorgchem.1c00995

**Published:** 2021-07-13

**Authors:** Henrik Braband, Michael Benz, Bernhard Spingler, Jeanet Conradie, Roger Alberto, Abhik Ghosh

**Affiliations:** † Department of Chemistry, University of Zurich, Zürich 8057, Switzerland; ‡ Department of Chemistry, UiT—The Arctic University of Norway, Tromsø N-9037, Norway; § Department of Chemistry, University of the Free State, P.O. Box 339, Bloemfontein 9300, South Africa

## Abstract

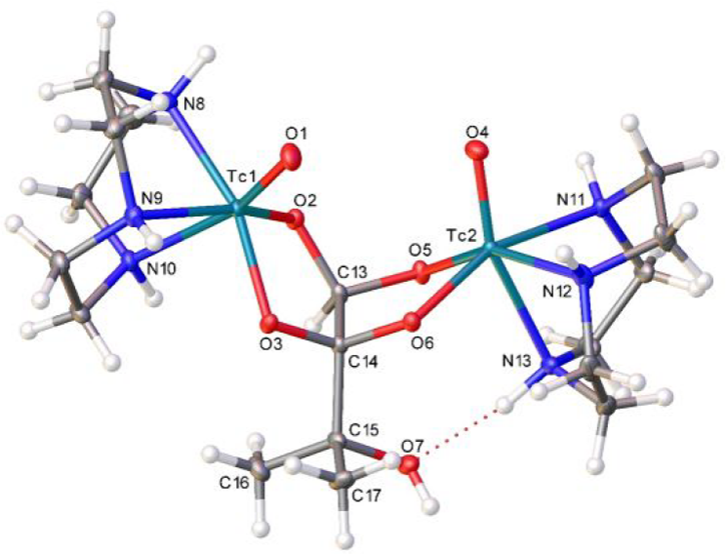

The difference in [3 + 2] cycloaddition reactivity between *fac*-[MO_3_(tacn)]^+^ (M = Re, ^99^Tc; tacn = 1,4,7-triazacyclononane) complexes has been reexamined with a selection of unsaturated substrates including sodium 4-vinylbenzenesulfonate, norbornene, 2-butyne, and 2-methyl-3-butyn-2-ol (2MByOH). None of the substrates was found to react with the Re cation in water at room temperature, whereas the ^99^Tc reagent cleanly yielded the [3 + 2] cycloadducts. Interestingly, a bis-adduct was obtained as the sole product for 2MByOH, reflecting the high reactivity of a ^99^TcO-enediolato monoadduct. On the basis of scalar relativistic and nonrelativistic density functional theory calculations of the reaction pathways, the dramatic difference in reactivity between the two metals has now been *substantially* attributed to differences in relativistic effects, which are much larger for the 5d metal. Furthermore, scalar-relativistic Δ*G* values were found to decrease along the series propene > norbornene > 2-butyne > dimethylketene, indicating major variations in the thermodynamic driving force as a function of the unsaturated substrate. The suggestion is made that scalar-relativistic effects, consisting of greater destabilization of the valence electrons of the 5d elements compared with those of the 4d elements, be viewed as a new design principle for novel ^99m^Tc/Re radiopharmaceuticals, as well as more generally in heavy-element coordination chemistry.

## Abstract 2

Room temperature cycloaddition reactivity of *fac*-[^99^TcO_3_(tacn)]^+^ (tacn = 1,4,7-triazacyclononane) with a variety of unsaturated substrates and the lack of such reactivity for *fac*-[ReO_3_(tacn)]^+^ appears largely attributable to much stronger relativistic effects for Re relative to Tc, based on relativistic density functional theory calculations.

## Introduction

Technetium-99m, a metastable nuclear isomer of technetium-99, is the most commonly used radioisotope in medicine, and the demand for ^99m^Tc radiopharmaceuticals with novel biodistribution properties is considerable.^1−4^ A common early step toward the development of these products involves model chemistries with ^99^Tc and Re. Although the two elements are chemically very similar, they exhibit quantitative differences in reactivity, reflecting the somewhat greater stability (and lower reduction potentials) of the higher oxidation states of Re. In a seminal finding, Pearlstein and Davison in the 1980s showed that *fac*-[^99^Tc^VII^O_3_]^+^ complexes undergo [3 + 2] cycloadditions with olefins to yield ^99^Tc^V^O diolate derivatives.^5^ The analogous Re^V^O-diolate species, in contrast, were found to be unstable, undergoing the opposite reaction when thermalized. We built on this finding to develop *fac*-[^99m^Tc^VII^O_3_]^+^ complexes as aqueous-phase labeling agents for olefins.^6−8^ The factors underlying the difference in reactivity between the two group 7 elements, however, have remained obscure. Physicochemical measurements at the Tromsø laboratory on analogous pairs of 4d and 5d metallocorroles,^9−11^ including those involving Mo^12^/W,^13^^99^Tc^V^O^14^/Re^V^O,^15^ Ru^VI^N^16^/Os^VI^N,^17^ and Ag/Au,^18,19^ suggested that relativistic effects might partly explain the difference in cycloaddition reactivity between ^99m/99^Tc and Re.^20^

Unfortunately, little is known about the importance of relativistic effects for transition-metal reactivity.^21−23^ For most of the 20th century, relativistic effects were not considered important for chemistry. Indeed, in 1929, Paul Dirac asserted that the only imperfections remaining in quantum mechanics “give rise to difficulties only when high-speed particles are involved, and are therefore of no importance in the consideration of atomic and molecular structure and ordinary chemical reactions in which it is, indeed, usually sufficiently accurate if one neglects relativity variation of mass and velocity and assumes only Coulomb forces between the various electrons and atomic nuclei.”^24^ This view started changing only in the 1970s.^25,26^ Today the importance of relativistic effects is well recognized for the static properties of sixth- and seventh-period elements.^27^ Relativity thus accounts for such well-known effects as the liquid state of Hg^28^ and the yellow color of elemental Au^29^ and Cs as well as a host of less well-known effects in heavy-element chemistry.^30−33^

## Results and Discussion

### Synthetic and Reactivity Studies

**Scheme 1:**
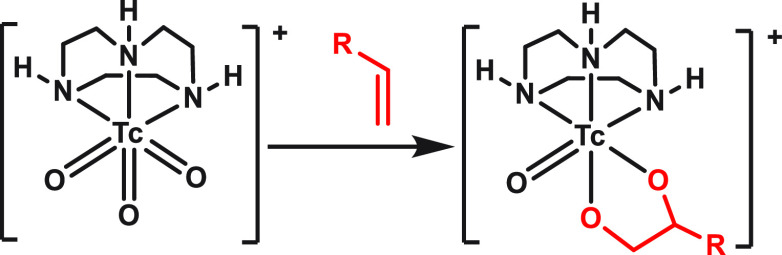
Cycloaddition of [^99^TcO_3_(tacn)]^+^ with Alkenes

With the above as the backdrop, we chose to perform a comparative study of *fac*-[MO_3_(tacn)]^+^ (M = Re, ^99^Tc; tacn = 1,4,7-triazacyclononane) complexes with respect to their [3 + 2] cycloaddition reactivity with a selection of unsaturated substrates including sodium 4-vinylbenzenesulfonate, norbornene, 2-butyne, and 2-methyl-3-butyn-2-ol (2MByOH; Scheme 1). Because we already knew from our recent work that *fac*-[^99^TcO_3_(tacn)]^+^ reacts with a broad range of olefins to yield ^99^TcO-diolate products, we focused here particularly on complexes of the type *fac*-[ReO_3_(tacn)]X (X = Cl, BPh_4_).^34^ We verified that the Re complexes do not react with olefins and alkynes, as indeed was expected from Pearlstein and Davison’s original observations.^6^

**Scheme 2:**
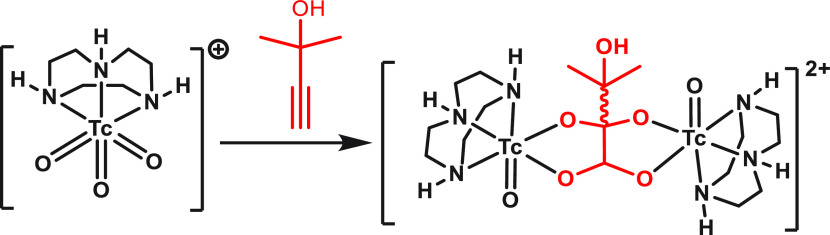
Double [3 + 2] Cycloaddition of Two *fac*-[^99^TcO_3_(tacn)]^+^ Cations with 2MByOH (Showing One of the Two Diastereomers Formed)

Because alkynes had not been examined as substrates until now, we chose to examine the interaction of the water-stable complex *fac*-[^99^TcO_3_(tacn)]Cl^8^ with the water-soluble propargylic alcohol 2MByOH. After the addition of 2 equiv of the propargylic alcohol to an aqueous solution of *fac*-[^99^TcO_3_(tacn)]Cl, a quick color change was observed from yellow to green. After stirring for 2 h at room temperature, the dinuclear complex [{^99^Tc(O)O_2_(tacn)}_2_(2MByOH)]Cl_2_ was isolated as the sole product following removal of all volatiles under high vacuum. No mononuclear intermediate was detected by either high-performance liquid chromatography (HPLC) or NMR. This finding suggests that the expected ^99^TcO-enediolate intermediate acts as a highly reactive substrate for a second equiv of *fac*-[^99^TcO_3_(tacn)]^+^ to yield the observed bis-adduct (Scheme 2).

**Scheme 3:**
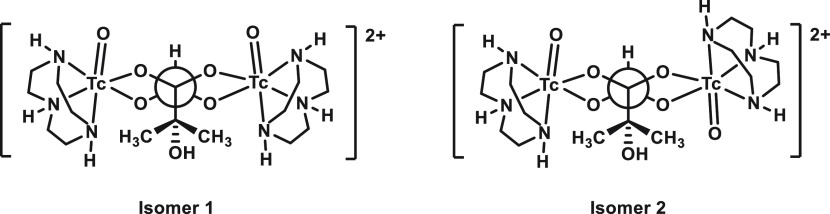
Observed Isomers of [{^99^Tc^V^(O)O_2_(tacn)}_2_(2MByOH)]Cl_2_

**Figure 1:**
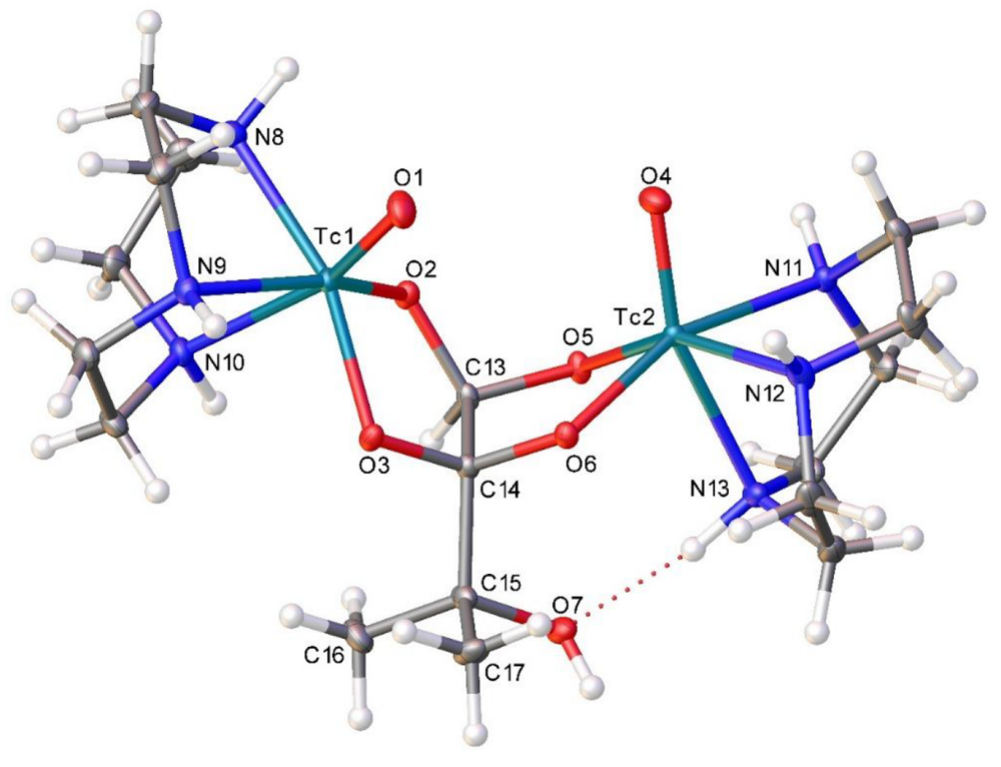
Thermal ellipsoid (50% probability) plot for [{^99^Tc^V^(O)O_2_(tacn)}_2_(2MByOH))Br_2_. Bromide ions and water molecules have been omitted for clarity. Selected bond distances (Å) and angles (deg): Tc1–O1 1.661(3), Tc1–O2 1.926(3), Tc2–O4 1.665(3), Tc2–O5 1.946(3), Tc1–N8 2.163(4), Tc1–N9 2.175(4), Tc1–N10 2.295(4), Tc2–N11 2.185(3), Tc2–N12 2.147(4), Tc2–N13 2.250(4); O1–Tc1–O2 112.86(16), O2–Tc1–O3 81.73(12), O4–Tc2–O5 108.23(16), O5–Tc2–O6 81.42(12), O2–C13–O5 107.6(3), O3–C14–O6 108.8(3).

The Fourier transform infrared spectrum of [{^99^Tc^V^(O)O_2_(tacn)}_2_(2MByOH)]Cl_2_ was found to exhibit a ν_Tc=O_ band at 967 cm^–1^, considerably upshifted relative to that in [^99^TcO(tacn)(eg)]^+^ (949 cm^–1^; eg = ethane-1,2-diolato).^35^ Given that two symmetry-nonequivalent addition modes are conceivable for the second equiv of *fac*-[^99^TcO_3_(tacn)]^+^, ^1^H and ^13^C NMR spectroscopy of [{^99^Tc^V^(O)O_2_(tacn)}_2_(2MByOH)]Cl_2_ understandably indicated the formation of two diastereomers in a 2:1 ratio (Scheme 3).^36^ Slow evaporation of an aqueous solution of the product in the presence of excess KBr led to crystallization of the major diastereomer of [{^99^Tc^V^(O)O_2_(tacn)}_2_(2MByOH)]Br_2_ (isomer 1 in Scheme 3). Single-crystal X-ray diffraction analysis (Table 1 and Figure 1) revealed an intramolecular N4–H···O7 hydrogen bond, which, along with less overall steric crowding, appears to be responsible for the formation of isomer 1 as the major product. In contrast to the [3 + 2] cycloadducts of *fac*-[^99^TcO_3_(tacn)]^+^ with alkenes, slow decomposition of isomer 1 of the bisadduct (formation of ([TcO_4_]^−^) was observed over days.

**Table 1:** Crystal Data and Structure Refinement for [{^99^Tc^V^(O)O_2_(tacn)}_2_(2MByOH)]Br_2_·2.2H_2_O

empirical formula	C_17_H_42_Br_2_N_6_O_9.20_Tc_2_
diffractometer	Xcalibur, Ruby diffractometer
wavelength (Å)	0.71073
fw	833.58
cryst syst	monoclinic
space group	*P*2_1_/*c*
*a* (Å)	16.5494(7)
*b* (Å)	13.3352(5)
*c* (Å)	14.509(2)
α (deg)	90
β (deg)	114.955(11)
γ (deg)	90
volume (Å^3^)	2903.1(6)
*Z*	4
density (calcd) (g cm^–3^)	1.907
temperature (K)	183.1
abs coeff (mm^–1^)	3.758
*F*(000)	1662
cryst size (mm^3^)	0.234 × 0.145 × 0.075
cryst description	green block
θ range for data collection (deg)	2.715–30.508
index ranges	–23 ≤ *h* ≤ 23, −19 ≤ *k* ≤ 19, −19 ≤ *l* ≤ 20
reflns collected	41201
indep reflns	8809 [*R*(int) = 0.0396)
reflns obsd	7686
criterion for observation	*I* > 2(*I*)
completeness to θ = 25.242° (%)	99.0
abs corrn	semiempirical from equivalents
max and min transmn	1.000 and 0.789
data/restraints/param	8809/6/362
GOF on *F*^2^	1.054
final *R* indices [*I* > 2σ(*I*)]	R1 = 0.0469, wR2 = 0.1202
*R* indices (all data)	R1 = 0.0550, wR2 = 0.1250
largest diff peak and hole (e Å^–3^)	2.533 and −2.461
CCDC	2071332

### Theoretical Modeling

Relativistic and nonrelativistic density functional theory (DFT) calculations (typically with large all-electron STO-TZ2P basis sets; see Experimental Section for details) were used to investigate the [3 + 2] cycloaddition of the cationic complexes [MO_3_(tacn)]^+^ (M = Tc, Re) with four different olefins, namely, propene, dimethylketene, 2-butyne, and norbornene, in acetonitrile (MeCN) as a solvent (Table 2). Relativity was taken into account either via effective core potentials (ECPs) or with a scalar-relativistic treatment with the zeroth-order regular approximation (ZORA). Two-component spin–orbit relativistic calculations were undertaken in a few cases as random checks on the quality of the ECP and scalar-relativistic results; the latter results were indeed found to be adequate, with minimal differences relative to the spin–orbit calculations. The data in Table 1 led to the following conclusions.

**Table 2:** Scalar-Relativistic and Nonrelativistic DFT Energetics (eV) for Different Substrates in CH_3_CN

		B3LYP_scalar_^a^		B3LYP_nrel_^a^		PBE0_scalar_^a^		PBE0_nrel_^a^		OPBE0_scalar_^a^		PBE-D2_ECP_^b^	
substrate	metal	Δ*G*	Δ*G*^⧧^	Δ*G*	Δ*G*^⧧^	Δ*G*	Δ*G*^⧧^	Δ*G*	Δ*G*^⧧^	Δ*G*	Δ*G*^⧧^	Δ*G*	Δ*G*^⧧^
propene	Tc	–0.38	0.83	–0.77	1.09	–0.91	1.13	–1.31	0.98	–0.71	1.46	–0.48	0.59
	Re	1.28	1.86	–0.81	1.10	0.33	1.69	–1.38	0.98	0.54	2.01	0.55	1.09
dimethylketene	Tc	–1.44	1.28	–1.83	1.17	–2.02	1.20	–2.42	1.10	–1.77	1.62	–1.47	0.50
	Re	–0.21	1.91	–1.74	1.05	–0.76	1.77	–2.41	0.92	–0.50	2.32	–0.41	0.93
2-butyne	Tc	–1.20	1.35	–1.62	1.26	–1.76	1.25	–2.18	1.14	–1.58	1.61	–1.35	0.70
	Re	–0.21	1.91	–1.50	1.25	–0.48	1.74	–2.10	1.09	–0.30	2.07	–0.21	1.11
norbornene	Tc	–0.73	1.10	–1.14	0.92	–1.25	0.98	–1.67	0.83	–1.00	1.35	–0.96	0.32
	Re	0.48	1.67	–1.20	0.80	–0.02	1.49	–1.77	0.66	0.24	1.87	0.07	0.73

a Obtained with *ADF*.

b Obtained with *Gaussian*.

**Figure 2:**
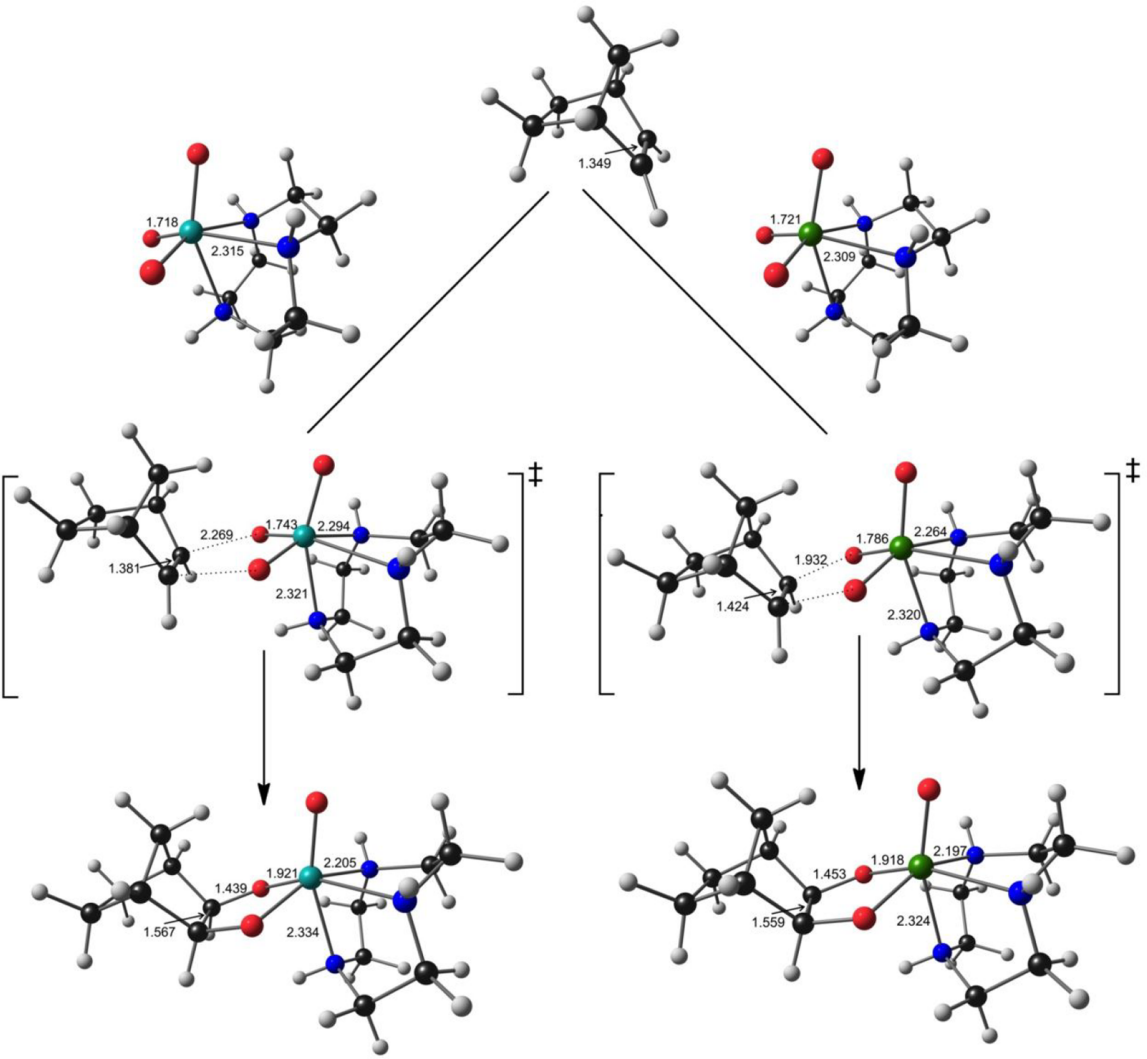
Ball-and-stick diagrams, with key distances (Å), for the optimized PBE-D2_ECP_ stationary points for the [3 + 2] cycloaddition of [MO_3_(tacn)]^+^ and norbornene. M = Tc (left), Re (right).

Relativistic calculations indicate dramatically lower (in an algebraic sense) reaction free energies (Δ*G*) and free energies of activation (Δ*G*^⧧^) for Tc than for Re, consistent with the experimentally observed difference in reactivity between the two metals. These translate to substantially “earlier” transition states for Tc than for Re; in other bonds, key bonds affected by the reaction are rather similar in length to the starting materials for the Tc reactions compared with the Re reactions (Figure 2). In sharp contrast, nonrelativistic calculations (B3LYP_nrel_ and PBE0_nrel_ in Table 2) indicate similar Δ*G* and Δ*G*^⧧^ values for the two metals. The fact that these generalizations hold regardless of the exchange-correlation functional and the organic substrate indicates that the difference in reactivity between the two metals is largely a relativistic effect.

The above interpretation is supported by computations of the adiabatic electron affinities (EAs) for the M(VII) d^0^ complexes MeTc^VII^O_3_ and MeRe^VII^O_3_ (Me = methyl). At the scalar relativistic level, the B3LYP values are 3.44 and 2.79 eV, respectively, i.e., the EA of the Tc(VII) complex is 650 meV higher than that of the Re(VII) complex. The scalar-relativistic PBE0 values are similar, 3.31 and 2.65 eV, as are the PBE-D2_ECP_ values, 3.67 and 3.02 eV. At the nonrelativistic level, the B3LYP EAs are 3.64 and 3.32 eV, while the PBE0 EAs are 3.51 and 3.20 eV, respectively, which translates to a difference of just over 300 meV between the two metals. These results prove that the difference in the EAs or reduction potentials between the Tc(VII) and Re(VII) species is substantially ascribable to the relativistic destabilization of the Re 5d orbitals relative to the Tc 4d orbitals. Much the same considerations should apply to the cycloaddition reaction of interest in this study because it also involves a reduction, albeit a two-electron one, of the M(VII) centers.

Another key observation from Table 2 is that the Δ*G* values, which decrease along the series propene > norbornene > 2-butyne > dimethylketene, reflect dramatic variations in the thermodynamic driving force as a function of the olefinic substrate. In fact, for propene, all of the relativistic methods yield positive Δ*G* values, consistent with the experimental observation that simple, unstrained olefins do not react with cationic [Re^VII^O_3_]^+^ reagents at room temperature.^37^ Interestingly, much smaller variations are observed among the Δ*G*^⧧^ values for the four substrates. Again, for Re, the calculations generally indicate the highest Δ*G*^⧧^ value for propene and lower values for dimethylketene and norbornene.

The above calculations are far from perfect. While the Δ*G* values are moderately consistent across different functionals (for the relativistic calculations), the Δ*G*^⧧^ values exhibit much wider variations. Of the different functionals examined, PBE-D2_ECP_ appears to yield the lowest, and probably most realistic, Δ*G*^⧧^ values, which has also been observed in a DFT study of Ir-catalyzed reactions.^38^ Overall, our results underscore the need for substantial additional benchmarking of different functionals vis-à-vis transition-metal-mediated redox reactions, especially for 4d and 5d elements.

## Conclusion

In earlier studies of metalloporphyrin-type compounds,^9–11^ we concluded that the difference in redox potential between analogous 4d and 5d complexes is *largely* attributable to scalar relativistic effects, much as calculated for Δ*G* and Δ*G*^⧧^ values in the present study. The greater relativistic destabilization of the valence electrons of the 5d elements compared with those of the 4d elements thus may be viewed as a reliable design principle for novel ^99m^Tc radiopharmaceuticals, as well as more generally in heavy-element coordination chemistry. In other words, higher-valent technetium species such as pertechnetate or *fac*-[^99/99m^TcO_3_]^+^ derivatives should be much more easily reduced (i.e., accept electrons in their 4d orbitals) than isoelectronic Re species (where electrons would be added to 5d orbitals). This prediction—in this case, a postdiction—is nicely illustrated by the facile synthesis of ^99(m)^Tc(I) organometallic^39^ compounds via the reduction of pertechnetate, the analogous synthesis of Re(I) organometallics being far less facile. We look forward to seeing additional applications of relativity as a design principle in the synthesis of new classes of heavy/element coordination compounds.

## Experimental Section

### Instrumental Methods

IR spectra were measured as KBr pellets on a PerkinElmer BXII spectrometer. ^1^H and ^13^C NMR were recorded on a Bruker AV2-500 500-MHz spectrometer. Reactivity studies with Re compounds were performed on a Waters Acquity UPLC System coupled to a Bruker Daltonics HCTTM electrospray ionization mass spectrometer, using an Acquity UPLC BEH C18 1.7 μm (2.1 × 50 mm) column. Ultraperformance liquid chromatography (UPLC) solvents were formic acid (0.1% in Millipore water) (solvent A) and UPLC-grade MeCN (solvent B). Applied UPLC gradient: 0–0.5 min, 95% A and 5% B; 0.5–4.0 min, linear gradient from 95% A and 5% B to 0% A and 100% B; 4.0–5.0 min, 0% A and 100% B. The flow rate was 0.6 mL min^–1^. Detection was performed at 250 and 480 nm (DAD). Reactivity studies with Tc compounds were performed on a Merck Hitachi LaChrom L7100 pump coupled to a Merck Hitachi LaChrom L7200 tunable UV detector. The detection of radioactive ^99^Tc complexes was performed with an equipped Berthold LB508 radiodetector. Separations were achieved on a Macherey-Nagel C_18_ reversed-phase column (EC-250/3 Nucleosil 100-5 C18), using a gradient of triethylamine phosphate (TEAP)/MeCN as the eluent, with a flow rate of 0.5 mL min^–1^. TEAP method: *t* = 0–3 min, 100% TEAP; 3–3.1 min, 100–75% TEAP; 3.1–9 min, 75% TEAP; 9–9.1 min, 75–66% TEAP, 9.1–12 min, 66% TEAP; 12–12.1 min, 66–0% TEAP, 15–15.1 min, 0–100% TEAP; 15.1–18 min, 100% TEAP.

### Synthetic and Reactivity Studies

***Caution!**^99^Tc is a weak β emitter. All experiments were performed in laboratories approved for working with low-level radioactive materials.*

[^99^TcO_3_(tacn)]Cl was prepared as previously reported.^40^ Double-distilled water (dd-water) was used throughout. All chemicals were of reagent-grade quality or higher and were obtained from commercial suppliers.

#### Synthesis of [ReO_3_(tacn)][ReO_4_].^41^

Dirhenium heptoxide (520 mg, 1.1 mmol) was dissolved in dry tetrahydrofuran (THF; 5.0 mL). A solution of 1,4,7-triazacyclononane (125 mg, 0.96 mmol) in dry THF (1.0 mL) was added, and the resulting mixture was stirred for 30 min at room temperature. The colorless precipitate was filtered off and dried under vacuum. Yield: 98% (589 mg, 0.96 mmol).

#### Synthesis of [ReO_3_(tacn)](BPh_4_)

The aforementioned complex [ReO_3_(tacn)][ReO_4_] (188 mg, 0.31 mmol) was dissolved in distilled water (10 mL). A solution of sodium tetraphenylborate (210 mg, 0.61 mmol) dissolved in water (5 mL) was added, and the resulting mixture was stirred for 30 min at room temperature. The product precipitated as a pale-gray solid and was filtered off. Yield: 59% (121 mg, 0.18 mmol). Analytical data are in agreement with the literature.

#### Synthesis of [ReO_3_(tacn)]Cl

DOWEX-1 anion-exchange resin in chloride form (1000 mg) was washed with dd-water until the washings showed a pH of 7.0. The resin was then added to a solution of [ReO_3_(tacn)][ReO_4_] (183 mg, 0.3 mmol) in water (5.0 mL), and the suspension was stirred for 30 min at room temperature. The resin was filtered off, and [ReO_3_(tacn)]Cl was isolated by evaporation of the solvent under high vacuum. The successful exchange of [ReO_4_]^−^ by Cl^–^ was proven by IR and electrospray ionization mass spectrometry (negative mode). Yield: 66% (79 mg, 0.20 mmol). Analytical data are in agreement with the literature.^42^

#### Reactions of [ReO_3_(tacn)](BPh_4_) in MeCN with Alkenes and Alkynes

To a solution of [ReO_3_(tacn)](BPh_4_) (36 mg, 0.05 mmol) in MeCN (3.0 mL) was added the olefin or alkyne of interest (0.5 mmol), and the reaction mixture was stirred for 2 h at room temperature, followed by UPLC–MS analysis. If no reaction was observed, the temperature was raised to 85 °C for 2 h, and the reaction mixture was again analyzed by UPLC–MS. We found no evidence for the formation of a [3 + 2] cycloadduct for either norbornene or 2-butyne.

#### Reactions of [ReO_3_(tacn)]Cl in Water with Alkenes and Alkynes

To a solution of [ReO_3_(tacn)]Cl (18 mg, 0.05 mmol) dissolved in dd-water (2.0 mL) was added a water-soluble olefin or alkyne (0.5 mmol), and the reaction mixture was stirred for 2 h at room temperature, followed by UPLC–MS analysis. If no reaction was observed, the temperature was raised to 85 °C for 2 h, and the reaction mixture was again analyzed by UPLC–MS. We found no evidence for the formation of a [3 + 2] cycloadduct for either 2MByOH or sodium 4-vinylbenzenesulfonate.

#### Synthesis of [{^99^Tc(O)O_2_(tacn)}_2_(2MByOH)]Cl_2_

To a yellow solution of [^99^TcO_3_(tacn)]Cl (6.23 mg, 0.02 mmol) in dd-water (1.0 mL) was added 2MByOH (4 μL, 0.04 mmol), resulting in a rapid color change to green. After stirring for 2 h at room temperature, the solvent and other volatiles were removed under high vacuum, affording [{^99^Tc(O)O_2_(tacn)}_2_(2MByOH)]Cl_2_ in quantitative yield. IR [cm^–1^]: 3456s, 3412s, 3120m, 2991w, 2913w, 2845w, 2050w, 1637s, 1619s, 1541w, 1488w, 1455w, 1423w, 1381w, 1356w, 1286w, 1264w, 1230w, 1174w, 1110w, 1064m, 1014m, 967s, 931m, 847w, 837m, 802w, 746w, 716w, 676w, 621w, 601w, 565w, 525w, 467w, 436w. ^1^H NMR (500 MHz, D_2_O): δ 8.11 (s, CH isomer 1, 1 H), 7.58 (s, CH isomer 2, 1 H), 3.77–2.20 (m, tacn, 36 H), 1.60 (s, CH_3_ isomer 1, 6 H), 1.45 (s, CH_3_ isomer 2, 3 H), 1.24 (s, CH_3_ isomer 2, 3 H). ^13^C NMR (125 MHz, D_2_O): δ 129.21 (O_2_CRR′, 1 C), 123.94 (CH isomer 2, 1 C), 120.07 (CH isomer 1, 1 C), 57.94–45.21 (tacn, 6 C), 28.02 (CH_3_ isomer 2, 1 C), 26.98 (CH_3_ isomer 1, 2 C), 25.07 (CH_3_ isomer 2, 1 C). See Scheme 3 for a definition of isomers 1 and 2.

Crystals of [{^99^Tc(O)O_2_(tacn)}_2_(2MByOH)]Br_2_ suitable for single-crystal X-ray diffraction analysis were obtained by slow evaporation of an aqueous solution of the product in the presence of excess KBr.

### X-ray Structure Analysis

Crystallographic data were collected at 183(2) K with Mo Kα radiation (λ = 0.7107 Å) monochromatized with graphite on an Oxford Diffraction Xcalibur system with a Ruby detector. Suitable crystals were covered with oil (Infineum V8512, formerly known as Paratone N), mounted atop a glass fiber, and immediately transferred to the diffractometer. The *CrysAlisPro*(43) program suite was used for data collection, semiempirical absorption correction, and data analysis. The structure was solved with direct methods using *SIR97*(44) and refined by full-matrix least-squares methods on *F*^2^ with *SHELXL-2018*(45) using the *Olex2* GUI.^46^ The refinement was done with anisotropic thermal parameters for all non-H atoms, unless otherwise indicated. The positions of the H atoms were calculated using the “riding atom” option in *SHELXL-2018*. More details on data collection and structure calculations are given in Table 1 and in the crystallographic information file.

### Computational Methods

The majority of DFT calculations (including full geometry optimizations in the presence of a solvent) were carried out with the *ADF 2018* program system.^47^ Relativistic effects were taken into account with the ZORA^48^ method, applied both as a scalar correction and with spin–orbit coupling at the two-component level. A parallel set of calculations were carried out with the same basis set but with a nonrelativistic Hamiltonian. Specially optimized all-electron ZORA STO-TZ2P basis sets were used throughout. A variety of exchange-correlation functionals were tested, including OLYP,^49,50^ B3LYP,^51,52^ PBE0,^53,54^ and OPBE0.^55^ The potential influence of dispersion corrections was examined, and, in general, they did not make a significant difference. Our results therefore generally refer to the pristine functionals. Zero-point energy and thermal corrections (vibrational, rotational, and translational) were made to the electronic energies in the calculation of the thermodynamic parameters. Enthalpies (*H*) and Gibbs free energies (*G*) were calculated from
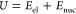
1
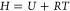
2
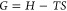
3where *U* is the gas-phase thermodynamic energy, *E*_el_ the total electronic energy, and *E*_nuc_ the nuclear internal energy (sum of the vibrational, rotational, and translational energies and the zero-point energy correction); *R* is the ideal gas constant, *T* the temperature, and *S* the entropy. *S* was calculated from the temperature-dependent partition function in *ADF* at 298.15 K. Solvent effects were taken into account with COSMO (conductor-like screening model),^56−58^ as implemented^59^ in *ADF*. The type of cavity used is Esurf,^60^ and the solvent used was MeCN (eps = 37.5; Rad = 2.76).

The *Gaussian 16* program system^61^ was used for the PBE-D2^62^^63^ calculations. The basis set was 6-311G(d,p) on all nonmetallic atoms and LANL2DZ with an ECP augmented with one f-polarization function on Re (0.869) and Tc (1.134). The polarizable continuum model (PCM)^64^ as its integral equation formalism variant (IEFPCM)^65^ was used for solvent (MeCN) calculations in *Gaussian*.

## Supporting Information Available

Selected spectra, additional crystallographic details, and optimized DFT coordinates (PDF)

## Accession Codes

CCDC 2071332 contains the supplementary crystallographic data for this paper. These data can be obtained free of charge via www.ccdc.cam.ac.uk/data_request/cif, or by emailing data_request@ccdc.cam.ac.uk, or by contacting The Cambridge Crystallographic Data Centre, 12 Union Road, Cambridge CB2 1EZ, UK; fax: +44 1223 336033.
