# Supplementary material for: Relativity as a Synthesis Design Principle: A Comparative Study of [3 + 2] Cycloaddition of Technetium(VII) and Rhenium(VII) Trioxo Complexes with Olefins
Source: Inorg Chem. 2021 Jul 13;60(15):11090–7. doi: 10.1021/acs.inorgchem.1c00995 (PMC8388117; doi:10.1021/acs.inorgchem.1c00995)
Supplement: Supplementary file 1 — ic1c00995_si_001.pdf [file ic1c00995_si_001.pdf]

Supporting Information

Relativity as a Synthesis Design Principle: A Comparative Study of (3 + 2) Cycloaddition of Technetium(VII)- and Rhenium(VII)-Trioxo Complexes with Olefins

Henrik Braband,[a] Michael Benz,[a] Bernhard Spingler,[a] Jeanet Conradie,*[,b,c]
Roger Alberto,*[,b] Abhik Ghosh*[,b]

Department of Chemistry, University of Zurich, 8057 Zürich, Switzerland; [b]Department of Chemistry, UiT – The Arctic University of Norway, N-9037 Tromsø, Norway; [c]Department of Chemistry, University of the Free State, P.O. Box 339, Bloemfontein, 9300, South Africa.

Contents **Page**

Contents	Page
A. Selected spectra	S2
B. Additional crystallographic details	S5
C. Additional DFT data	S7
D, E. Selected optimized coordinates	S8

A. Selected spectra

Note: The two diastereomers of [{$^{99}Tc^{V}(O)O_2(tacn)\}_2(2MByOH)]Cl_2$ could not be separated and individually characterized. The following spectra thus show a mixture of the two diastereomers.

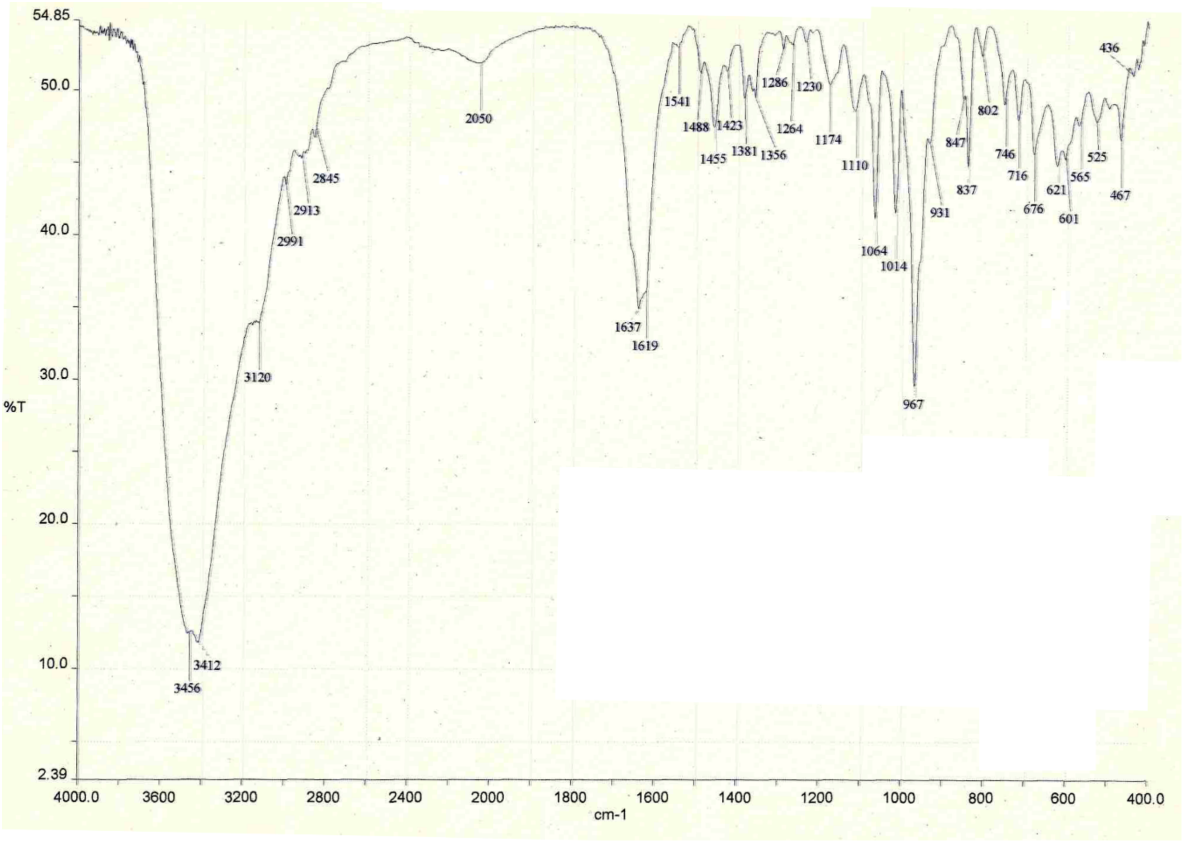

Figure S1. IR spectrum of [{$^{99}Tc^{V}(O)O_2(tacn)\}_2(2MByOH)]Cl_2$.

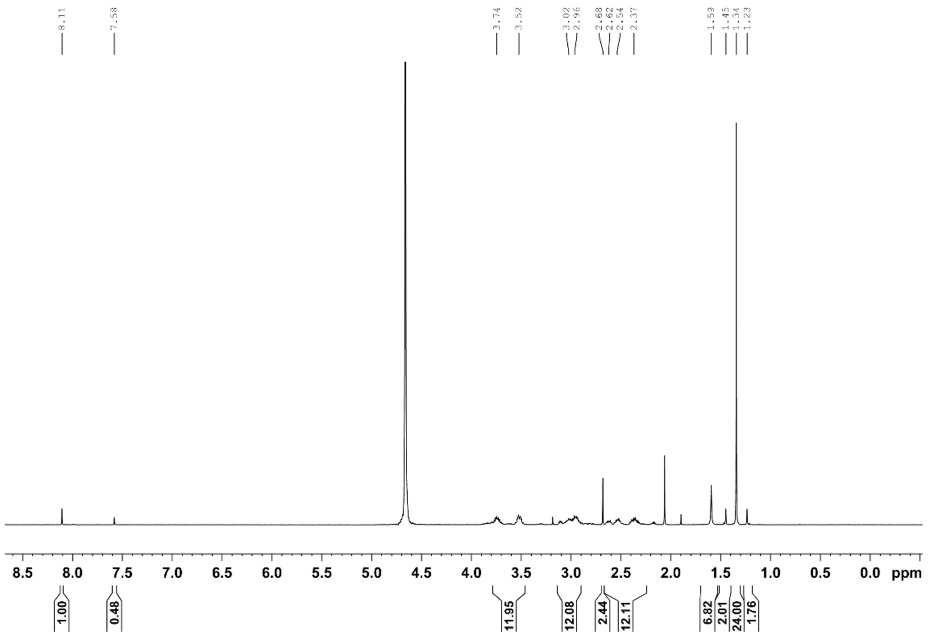

Figure S2. ^{1}H NMR spectrum of $[\{^{99}Tc^{V}(O)O_2(tacn)\}_2(2MByOH)]Cl_2$ (D_2O, 293 K). Signals at 2.68 and 1.34 ppm have been assigned to 2-methyl-3-butyn-2-ol.

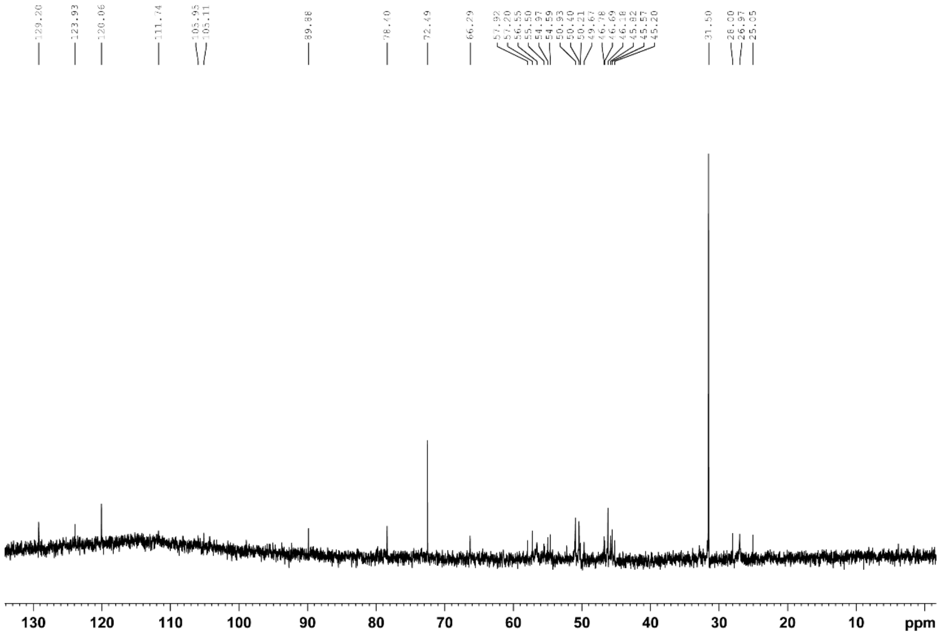

Figure S3. ^{13}C NMR spectrum of $[\{^{99}Tc^{V}(O)O_2(tacn)\}_2(2MByOH)]Cl_2$ (D_2O, 293 K). Signals at 89.88, 72.49, 66.29 and 31.50 ppm have been assigned to 2-methyl-3-butyn-2-ol.

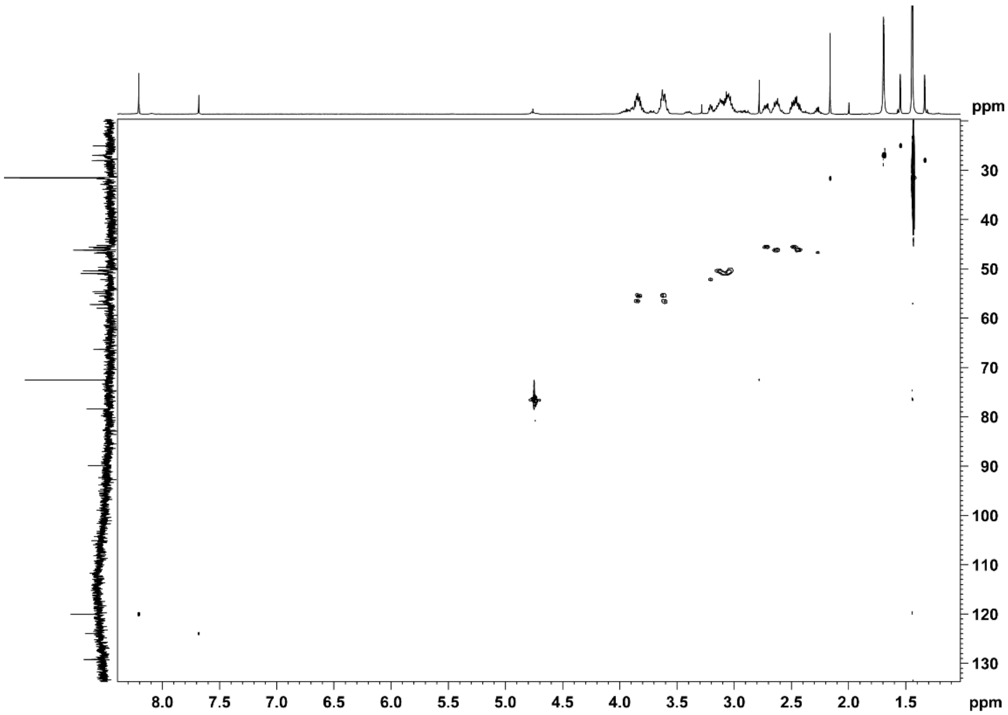

Figure S4. ^{13}C-^{1}H correlation spectrum of $[\{^{99}Tc^{V}(O)O_2(tacn)\}_2(2MByOH)]Cl_2$ (D_2O, 293 K).

B. Additional crystallographic details

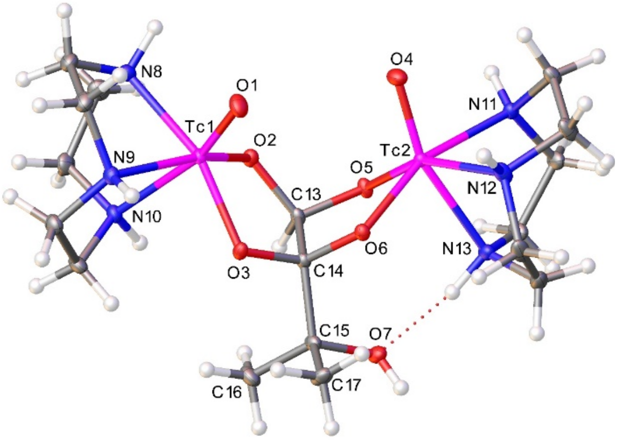

Figure S5. Thermal ellipsoid plot (50% probability) for [{$^{99}Tc^{V}(O)O_2(tacn)\}_2$-(2MByOH)]$Br_2$· 2.2($H_2O$).

Table S1. Bond lengths for [{$^{99}Tc^{V}(O)O_2(tacn)\}_2$(2MByOH)]$Br_2$· 2.2($H_2O$).

Tc1—N10	2.295 (4)
Tc1—N9	2.175 (4)
Tc1—N8	2.163 (4)
Tc1—O1	1.661 (3)
Tc1—O2	1.926 (3)
Tc1—O3	1.936 (3)
Tc2—N13	2.250 (4)
Tc2—N12	2.147 (4)
Tc2—N11	2.185 (3)
Tc2—O4	1.665 (3)
Tc2—O5	1.946 (3)
Tc2—O6	1.942 (3)
C13—C14	1.544 (5)

Table S2. Selected bond angles for $[\{^{99}Tc^{V}(O)O_2(tacn)\}_2\text{-}(2MByOH)]Br_2 \cdot 2.2(H_2O)$.

N9—Tc1—N10	74.80 (13)	N12—Tc2—N13	75.55 (16)
N8—Tc1—N10	75.04 (14)	N12—Tc2—N11	80.12 (14)
N8—Tc1—N9	79.99 (14)	N11—Tc2—N13	75.02 (13)
O1—Tc1—N10	162.07 (15)	O4—Tc2—N13	161.23 (15)
O1—Tc1—N9	89.61 (16)	O4—Tc2—N12	96.98 (17)
O1—Tc1—N8	93.83 (17)	O4—Tc2—N11	86.82 (15)
O1—Tc1—O2	112.86 (16)	O4—Tc2—O5	108.23 (16)
O1—Tc1—O3	107.08 (16)	O4—Tc2—O6	112.55 (14)
O2—Tc1—N10	82.37 (13)	O5—Tc2—N13	79.62 (14)
O2—Tc1—N9	157.17 (13)	O5—Tc2—N12	154.66 (15)
O2—Tc1—N8	93.97 (14)	O5—Tc2—N11	98.36 (13)
O2—Tc1—O3	81.73 (12)	O6—Tc2—N13	85.16 (13)
O3—Tc1—N10	83.72 (13)	O6—Tc2—N12	91.46 (13)
O3—Tc1—N9	95.91 (13)	O6—Tc2—N11	159.82 (13)
O3—Tc1—N8	158.73 (14)	O6—Tc2—O5	81.42 (12)
O2—C13—C14	108.5 (3)	O3—C14—C15	105.7 (3)
O5—C13—C14	110.6 (3)	O3—C14—O6	108.8 (3)
O5—C13—O2	107.6 (3)	O6—C14—C13	106.9 (3)
C13—C14—C15	117.3 (3)	O6—C14—C15	109.1 (3)
O3—C14—C13	108.8 (3)		

C. DFT data.

Table S3. Imaginary frequency for the transition state of the indicated reactions, obtained in CH_3CN with the indicated methods.

Reaction	$B3LYP_{scalar}$[a]	$B3LYP_{nrel}$[a]	$PBE0_{scalar}$[a]	$PBE0_{scalar}$[a]	$OPBE0_{scalar}$[a]	$PBE\text{-}D2_{ECP}$[b]
$[Re(tacn)O_3]^+$ + propene	-453.2	-382.6	-534.4	-423.7	-533.9	-426.8
$[Tc(tacn)O_3]^+$ + propene	-475.1	-331.9	-415.0	-363.6	-417.2	-289.4
$[Re(tacn)O_3]^+$ + ketene	-558.6	-390.4	-384.7	-419.2	-407.0	-360.4
$[Tc(tacn)O_3]^+$ + ketene	-487.2	-381.0	-287.3	-384.9	-440.5	-328.8
$[Re(tacn)O_3]^+$ + 2-butyne	-446.3	-351.0	-481.2	-395.5	-486.5	-365.7
$[Tc(tacn)O_3]^+$ + 2-butyne	-356.6	-325.9	-417.8	-366.2	-410.2	-262.9
$[Re(tacn)O_3]^+$ + norbornene	-405.7	-231.2	-436.6	-293.4	-438.8	-324.7
$Tc(tacn)O_3]^+$ + norbornene	-253.8	-189.1	-394.2	-232.9	-308.7	-73.72

[a] Obtained with ADF; [b] Obtained with Gaussian.

D. B3LYP/ZORA-STO-TZ2P optimized coordinates (Å)

(a) 2-butyne

C	-2.047750000	0.068221000	-2.258269000
C	-2.051166000	0.045843000	-3.716039000
C	-2.054342000	0.025616000	-4.916679000
C	-2.058070000	0.001650000	-6.374351000
H	-1.040424000	-0.013695000	-6.770375000
H	-1.444929000	0.896007000	-1.878472000
H	-1.635921000	-0.857835000	-1.851763000
H	-2.560728000	0.881684000	-6.781261000
H	-2.576116000	-0.882379000	-6.752231000
H	-3.059733000	0.183925000	-1.864125000

(b) dimethylketene

C	0.094019000	1.498629000	0.200791000
C	-0.354385000	0.066195000	0.010677000
C	-0.554904000	-0.714228000	1.042254000
C	-0.573762000	-0.452491000	-1.393971000
H	0.222587000	1.754656000	1.251757000
H	0.347294000	-0.374552000	-1.979131000
H	1.047298000	1.672405000	-0.306670000
H	-0.639074000	2.189114000	-0.227059000
H	-0.889037000	-1.495210000	-1.403921000
H	-1.341322000	0.136788000	-1.904372000
O	-0.732459000	-1.413388000	1.962006000

(c) norbornene

C	1.305551000	-0.553198000	1.804782000
C	1.316367000	-0.530661000	-0.449211000
C	1.325857000	-2.077450000	1.442022000
C	1.332590000	-2.062031000	-0.116875000
C	2.214987000	0.008845000	0.687753000
C	-0.037747000	-0.015161000	0.009748000
C	-0.043452000	-0.026528000	1.344005000
H	0.467524000	-2.604084000	1.856937000
H	0.476327000	-2.578337000	-0.548760000
H	1.583053000	-0.333720000	2.833265000
H	1.603805000	-0.290529000	-1.470318000
H	2.228010000	-2.547729000	1.836010000
H	2.236826000	-2.526326000	-0.513142000
H	2.277129000	1.096647000	0.698701000
H	3.219077000	-0.420770000	0.688047000
H	-0.871151000	0.203158000	-0.643773000
H	-0.882561000	0.177910000	1.995005000

(d) propene

C	-2.440625000	0.129464000	-0.924920000
C	-2.907222000	-0.120685000	-2.141750000
C	-2.946314000	-0.488761000	0.342473000
H	-1.626549000	0.841901000	-0.816228000
H	-2.148924000	-1.031408000	0.857772000
H	-2.496481000	0.363203000	-3.018469000
H	-3.301823000	0.278789000	1.035618000
H	-3.718453000	-0.821552000	-2.306779000
H	-3.765113000	-1.183172000	0.149839000

(e) $[Re(tacn)O_3]^+$

C	2.077929000	2.335108000	2.863501000
C	2.235691000	0.883179000	3.315482000
C	3.531611000	4.341959000	3.265399000
C	4.688837000	0.624149000	2.850805000
C	4.861460000	3.738160000	2.813807000
C	5.878715000	1.489019000	3.267117000
H	1.022438000	2.562781000	2.720868000
H	1.531231000	0.651662000	4.110950000
H	1.935621000	3.719766000	4.391930000
H	2.048297000	0.208616000	2.477898000
H	2.567000000	2.495948000	1.904575000
H	3.033034000	4.822999000	2.421650000
H	3.601474000	-0.180977000	4.413038000
H	3.694227000	5.086037000	4.041754000
H	4.279987000	0.961575000	1.900102000
H	4.743647000	3.207238000	1.871016000
H	5.015260000	-0.404818000	2.705423000
H	5.585964000	4.532434000	2.639625000
H	6.145811000	3.212047000	4.345335000
H	6.451421000	0.998907000	4.051295000
H	6.535115000	1.658569000	2.411457000
N	2.679100000	3.277263000	3.858035000
N	3.598938000	0.680737000	3.873815000
N	5.386300000	2.774168000	3.830092000
O	2.429195000	1.687874000	6.201316000
O	4.154662000	3.845180000	6.158877000
O	5.160666000	1.272386000	6.172418000
Re	3.907178000	2.262226000	5.561114000

(f) $[Re(tacn)O_3]^+$ + dimethylketene product

C	0.379004000	2.171297000	5.885038000
C	1.462806000	1.510793000	9.372540000
C	1.822190000	1.811536000	7.915400000
C	-0.349375000	-0.181803000	8.996074000
C	-0.424895000	0.882509000	6.042414000
C	-1.554006000	-0.124417000	8.063056000
C	-3.349656000	4.229822000	7.420133000
C	-3.427505000	5.672691000	7.928137000

C	-3.846871000	3.272420000	8.515123000
C	-4.157011000	4.037614000	6.139148000
H	0.225338000	0.040263000	6.271311000
H	0.520185000	-0.589539000	8.483086000
H	1.167951000	3.606796000	7.108004000
H	1.254848000	2.003570000	5.255406000
H	1.648288000	2.380937000	9.997477000
H	1.922315000	0.890276000	7.345847000
H	2.082421000	0.688192000	9.736673000
H	2.789868000	2.309858000	7.879460000
H	-0.232385000	1.231078000	10.493308000
H	-0.233353000	2.950651000	5.435137000
H	-0.561255000	-0.846264000	9.832159000
H	-0.924218000	0.644674000	5.103376000
H	-1.650765000	-1.065307000	7.516790000
H	-2.327226000	1.234237000	6.745387000
H	-2.466715000	0.038754000	8.633436000
H	-2.821372000	5.809003000	8.824210000
H	-3.063281000	6.349140000	7.155207000
H	-3.790099000	4.716313000	5.369557000
H	-4.077687000	3.014332000	5.766197000
H	-4.459117000	5.927477000	8.166534000
H	-5.208993000	4.245310000	6.326277000
N	0.006025000	1.185265000	9.506963000
N	0.783644000	2.674500000	7.234001000
N	-1.416015000	1.034925000	7.145306000
O	-0.248278000	3.984274000	9.551312000
O	-1.963780000	3.901433000	7.145281000
O	-2.840453000	2.524035000	9.063120000
O	-4.982882000	3.154245000	8.865850000
Re	-1.009413000	2.924558000	8.491827000

(g) $[Re(tacn)O_3]^+$ + 2-butyne product

C	0.087371000	1.990959000	6.054631000
C	0.107700000	-1.311466000	7.671206000
C	0.407638000	0.501889000	5.962717000
C	0.588189000	1.742290000	9.718755000
C	0.762390000	-0.619153000	8.867712000
C	0.936729000	2.418025000	8.392263000
C	-3.461496000	3.152681000	8.665009000
C	-3.695299000	2.880085000	7.378461000
C	-4.055700000	4.172911000	9.570472000
C	-4.635060000	3.506043000	6.409505000
H	0.377236000	0.180482000	4.922493000
H	0.858533000	-1.886945000	7.125011000
H	0.918962000	2.579284000	5.659467000
H	0.992965000	-1.360657000	9.631364000
H	1.224056000	3.453476000	8.573668000
H	1.414236000	0.299069000	6.324902000
H	1.490540000	1.573150000	10.309632000
H	1.706187000	-0.160921000	8.579526000

H	1.791551000	1.935024000	7.922124000
H	-0.097031000	2.362408000	10.293357000
H	-0.497533000	0.123283000	10.337885000
H	-0.670559000	-1.993152000	8.006458000
H	-0.724957000	3.228431000	7.469888000
H	-0.805696000	2.219901000	5.476050000
H	-1.169375000	-0.815552000	6.145153000
H	-3.276843000	4.796246000	10.016936000
H	-4.100710000	3.864020000	5.526045000
H	-4.596516000	3.691851000	10.389012000
H	-4.749306000	4.819818000	9.037289000
H	-5.164472000	4.345187000	6.856058000
H	-5.373984000	2.776852000	6.068734000
N	-0.124465000	0.454746000	9.452794000
N	-0.223014000	2.344466000	7.461412000
N	-0.557049000	-0.309420000	6.777860000
O	-2.494122000	2.336199000	9.255883000
O	-2.716210000	-0.554696000	8.582712000
O	-2.922170000	1.843077000	6.857444000
Re	-1.885332000	0.854887000	8.171232000

(h) $[Re(tacn)O_3]^+$ + norbornene product

C	0.024771000	-1.291971000	7.878091000
C	0.148791000	1.855065000	5.990000000
C	0.397620000	0.350510000	6.022417000
C	0.636996000	1.897834000	9.663516000
C	0.703163000	-0.531618000	9.021360000
C	1.017801000	2.434717000	8.284395000
C	-3.434236000	3.412148000	8.553102000
C	-3.710254000	2.955553000	7.090012000
C	-4.807711000	3.265772000	9.234976000
C	-5.210577000	2.606064000	7.105686000
C	-5.397929000	2.030626000	8.524402000
C	-5.702304000	4.399227000	8.678605000
C	-5.982182000	3.942710000	7.211353000
H	0.350363000	-0.052912000	5.011961000
H	0.757503000	-1.935459000	7.385261000
H	0.893919000	-1.220637000	9.843140000
H	1.008750000	2.367273000	5.551531000
H	1.357974000	3.466426000	8.373955000
H	1.395010000	0.131925000	6.400908000
H	1.530423000	1.746979000	10.273096000
H	1.670474000	-0.146122000	8.705801000
H	1.846842000	1.869762000	7.861889000
H	-0.022282000	2.597403000	10.174040000
H	-0.516575000	0.389654000	10.420127000
H	-0.596246000	3.244320000	7.292861000
H	-0.731619000	2.076437000	5.389845000
H	-0.777782000	-1.915948000	8.263021000
H	-1.231094000	-0.880056000	6.311010000
H	-3.060435000	4.436240000	8.624240000

H	-3.489444000	3.726702000	6.348087000
H	-4.739783000	3.233596000	10.320129000
H	-4.856293000	1.105477000	8.708724000
H	-5.216864000	5.374394000	8.731929000
H	-5.506229000	1.977061000	6.268789000
H	-5.653604000	4.673506000	6.471055000
H	-6.446235000	1.866471000	8.773994000
H	-6.624322000	4.466444000	9.254487000
H	-7.045508000	3.773593000	7.047717000
N	-0.129868000	0.626844000	9.511590000
N	-0.145371000	2.338165000	7.359961000
N	-0.600421000	-0.345259000	6.900259000
O	-2.420678000	2.563187000	9.132040000
O	-2.692790000	-0.385001000	8.770574000
O	-2.865625000	1.828293000	6.775084000
Re	-1.888409000	0.982460000	8.192878000

(i) $[Re(tacn)O_3]^+$ + propene product

C	0.087371000	1.990959000	6.054631000
C	0.107700000	-1.311466000	7.671206000
C	0.407638000	0.501889000	5.962717000
C	0.588189000	1.742290000	9.718755000
C	0.762390000	-0.619153000	8.867712000
C	0.936729000	2.418025000	8.392263000
C	-3.461496000	3.152681000	8.665009000
C	-3.695299000	2.880085000	7.378461000
C	-4.055700000	4.172911000	9.570472000
C	-4.635060000	3.506043000	6.409505000
H	0.377236000	0.180482000	4.922493000
H	0.858533000	-1.886945000	7.125011000
H	0.918962000	2.579284000	5.659467000
H	0.992965000	-1.360657000	9.631364000
H	1.224056000	3.453476000	8.573668000
H	1.414236000	0.299069000	6.324902000
H	1.490540000	1.573150000	10.309632000
H	1.706187000	-0.160921000	8.579526000
H	1.791551000	1.935024000	7.922124000
H	-0.097031000	2.362408000	10.293357000
H	-0.497533000	0.123283000	10.337885000
H	-0.670559000	-1.993152000	8.006458000
H	-0.724957000	3.228431000	7.469888000
H	-0.805696000	2.219901000	5.476050000
H	-1.169375000	-0.815552000	6.145153000
H	-3.276843000	4.796246000	10.016936000
H	-4.100710000	3.864020000	5.526045000
H	-4.596516000	3.691851000	10.389012000
H	-4.749306000	4.819818000	9.037289000
H	-5.164472000	4.345187000	6.856058000
H	-5.373984000	2.776852000	6.068734000
N	-0.124465000	0.454746000	9.452794000
N	-0.223014000	2.344466000	7.461412000

N	-0.557049000	-0.309420000	6.777860000
O	-2.494122000	2.336199000	9.255883000
O	-2.716210000	-0.554696000	8.582712000
O	-2.922170000	1.843077000	6.857444000
Re	-1.885332000	0.854887000	8.171232000

(j) $[Tc(tacn)O_3]^+$

C	2.060186000	1.504451000	13.010099000
C	2.987228000	3.793134000	13.453008000
C	3.276103000	0.689522000	13.452956000
C	4.300481000	4.439568000	13.009576000
C	5.721266000	1.030363000	13.012457000
C	5.822682000	2.492810000	13.450299000
H	1.383341000	1.650922000	13.854868000
H	1.518894000	0.984254000	12.222902000
H	1.741674000	3.205259000	11.910633000
H	2.234971000	4.564961000	13.611853000
H	2.983311000	-0.347922000	13.612044000
H	3.114232000	3.277835000	14.403364000
H	3.658709000	1.056108000	14.403811000
H	4.119585000	5.166723000	12.221051000
H	4.407635000	-0.094128000	11.910875000
H	4.766505000	4.953498000	13.852920000
H	5.318843000	2.644621000	14.403075000
H	5.929268000	0.373792000	13.860130000
H	5.932442000	3.865523000	11.908332000
H	6.442971000	0.816194000	12.227409000
H	6.868752000	2.756390000	13.602394000
N	2.506501000	2.802307000	12.444853000
N	4.375360000	0.769356000	12.445414000
N	5.201976000	3.404760000	12.443769000
O	2.832494000	1.294944000	10.146329000
O	3.733060000	3.887545000	10.144099000
O	5.527936000	1.809321000	10.144977000

(k) $[Tc(tacn)O_3]^+$ + dimethylketene product

C	0.364438000	2.176841000	5.884360000
C	1.448235000	1.521093000	9.373158000
C	1.806601000	1.825110000	7.915354000
C	-0.352747000	-0.181613000	8.997252000
C	-0.434896000	0.885710000	6.046430000
C	-1.558043000	-0.123391000	8.064373000
C	-3.328161000	4.220651000	7.416319000
C	-3.412565000	5.662844000	7.928814000
C	-3.831349000	3.261693000	8.512853000
C	-4.132689000	4.029803000	6.132767000
H	0.220347000	0.046926000	6.274451000
H	0.517268000	-0.584881000	8.481038000
H	1.153843000	3.618049000	7.101899000

H	1.236909000	2.011156000	5.248784000
H	1.631698000	2.391285000	9.998485000
H	1.910827000	0.903565000	7.346915000
H	2.078063000	0.704361000	9.734097000
H	2.775075000	2.322813000	7.883327000
H	-0.241127000	1.227083000	10.499824000
H	-0.252315000	2.953032000	5.435178000
H	-0.561991000	-0.852548000	9.829122000
H	-0.932806000	0.643662000	5.107297000
H	-1.654601000	-1.065539000	7.519301000
H	-2.333811000	1.241043000	6.754509000
H	-2.470047000	0.036646000	8.636540000
H	-2.812288000	5.798496000	8.828816000
H	-3.046669000	6.342841000	7.159916000
H	-3.766499000	4.711240000	5.365342000
H	-4.051143000	3.007717000	5.757707000
H	-4.446653000	5.912232000	8.161814000
H	-5.185134000	4.235417000	6.319824000
N	0.774252000	2.684122000	7.227561000
N	-0.001843000	1.181748000	9.513905000
N	-1.422838000	1.034758000	7.149285000
O	-0.246386000	3.965048000	9.545731000
O	-1.945863000	3.896443000	7.149399000
O	-2.831031000	2.518188000	9.067217000
O	-4.970528000	3.147892000	8.856003000
Tc	-1.015250000	2.933910000	8.500589000

(l) $[Tc(tacn)O_3]^+$ + 2-butyne product

C	0.084206000	-1.305328000	7.677396000
C	0.104131000	1.987816000	6.045269000
C	0.422045000	0.497051000	5.962383000
C	0.595899000	1.753609000	9.711396000
C	0.742816000	-0.615238000	8.874940000
C	0.952411000	2.417379000	8.380584000
C	-3.471286000	3.123456000	8.679567000
C	-3.700781000	2.859808000	7.383127000
C	-4.070851000	4.147269000	9.579916000
C	-4.637472000	3.506038000	6.422619000
H	0.406882000	0.174128000	4.921907000
H	0.830291000	-1.902783000	7.147485000
H	0.935517000	2.571134000	5.641203000
H	0.963430000	-1.360050000	9.638944000
H	1.255506000	3.449447000	8.557740000
H	1.424144000	0.294506000	6.337610000
H	1.496306000	1.587815000	10.307136000
H	1.694291000	-0.172563000	8.587276000
H	1.802124000	1.920260000	7.915737000
H	-0.086041000	2.383658000	10.279548000
H	-0.509153000	0.149247000	10.340589000
H	-0.698061000	3.242585000	7.452231000
H	-0.708022000	-1.970358000	8.013805000

H	-0.789055000	2.212694000	5.464824000
H	-1.168438000	-0.806778000	6.131762000
H	-3.295489000	4.780460000	10.018074000
H	-4.098937000	3.884051000	5.550216000
H	-4.603298000	3.666706000	10.404102000
H	-4.773474000	4.783090000	9.045445000
H	-5.172212000	4.333174000	6.884710000
H	-5.371835000	2.781668000	6.062677000
N	-0.123204000	0.470581000	9.457606000
N	-0.204412000	2.354878000	7.447635000
N	-0.553194000	-0.306547000	6.765971000
O	-2.500255000	2.326567000	9.260583000
O	-2.710473000	-0.512479000	8.559151000
O	-2.919455000	1.851307000	6.850691000
Tc	-1.877223000	0.876517000	8.159263000

(m) $[Tc(tacn)O_3]^+$ + norbornene product

C	0.008558000	-1.282494000	7.874242000
C	0.136734000	1.864095000	5.993222000
C	0.386048000	0.358720000	6.020450000
C	0.625330000	1.904107000	9.665046000
C	0.688953000	-0.521846000	9.017363000
C	1.004833000	2.439748000	8.284871000
C	-3.415911000	3.398286000	8.549225000
C	-3.699566000	2.931606000	7.084688000
C	-4.790710000	3.262394000	9.235258000
C	-5.204039000	2.593032000	7.111083000
C	-5.387940000	2.026200000	8.532915000
C	-5.680448000	4.396994000	8.677521000
C	-5.960969000	3.938673000	7.210784000
H	0.344283000	-0.039681000	5.007318000
H	0.742691000	-1.930267000	7.387883000
H	0.888856000	-1.215820000	9.833456000
H	0.996337000	2.375365000	5.551300000
H	1.349402000	3.470507000	8.375312000
H	1.383440000	0.140944000	6.400185000
H	1.519910000	1.761433000	10.276062000
H	1.654239000	-0.135357000	8.696480000
H	1.833986000	1.873437000	7.863787000
H	-0.035729000	2.603869000	10.173258000
H	-0.523991000	0.392529000	10.425323000
H	-0.613640000	3.246329000	7.301778000
H	-0.743437000	2.085388000	5.392660000
H	-0.793650000	-1.905948000	8.260718000
H	-1.242248000	-0.875209000	6.300777000
H	-3.041533000	4.423921000	8.609649000
H	-3.479116000	3.700047000	6.338886000
H	-4.717315000	3.234164000	10.320349000
H	-4.847441000	1.100560000	8.719012000
H	-5.193427000	5.371605000	8.730046000
H	-5.507873000	1.962407000	6.278314000

H	-5.619243000	4.662653000	6.469724000
H	-6.435590000	1.865632000	8.787879000
H	-6.602851000	4.466065000	9.252811000
H	-7.025712000	3.783138000	7.042351000
N	-0.136045000	0.632418000	9.518385000
N	-0.155804000	2.344102000	7.361360000
N	-0.611493000	-0.342199000	6.891230000
O	-2.411957000	2.553465000	9.127903000
O	-2.672694000	-0.385458000	8.781242000
O	-2.866983000	1.803347000	6.778043000
Tc	-1.908511000	0.974955000	8.197754000

(n) $[Tc(tacn)O_3]^+$ + propene product

C	0.213225000	0.440642000	5.961818000
C	0.687512000	-0.544071000	8.898515000
C	0.707586000	1.859130000	9.634180000
C	1.010530000	2.438946000	8.254152000
C	-0.003016000	1.951703000	6.004046000
C	-0.078168000	-1.257459000	7.779083000
C	-3.614054000	3.231588000	8.810623000
C	-3.660900000	3.047602000	7.287332000
C	-4.820248000	2.655179000	9.539895000
H	0.092812000	2.549926000	10.208511000
H	0.099948000	0.084125000	4.938725000
H	0.611465000	-1.900844000	7.226998000
H	0.842794000	2.459194000	5.532618000
H	0.927177000	-1.266629000	9.677868000
H	1.227463000	0.189230000	6.268513000
H	1.374602000	3.461247000	8.359822000
H	1.635459000	1.679708000	10.182472000
H	1.636281000	-0.161632000	8.528398000
H	1.803698000	1.878117000	7.762292000
H	-0.419793000	0.340434000	10.408710000
H	-0.647911000	3.303314000	7.400718000
H	-0.864515000	-1.880990000	8.196629000
H	-0.908531000	2.212922000	5.460506000
H	-1.414191000	-0.782446000	6.296214000
H	-3.189475000	3.889271000	6.769022000
H	-3.511725000	4.291575000	9.057234000
H	-4.683304000	2.737184000	10.617623000
H	-4.685771000	2.961181000	6.921387000
H	-4.972871000	1.604558000	9.287165000
H	-5.720458000	3.207981000	9.267011000
N	-0.083870000	0.606302000	9.488743000
N	-0.206345000	2.391396000	7.402518000
N	-0.742500000	-0.277766000	6.865098000
O	-2.399711000	2.586238000	9.264302000
O	-2.674275000	-0.326499000	8.944503000
O	-2.955178000	1.849368000	6.931094000
Tc	-1.930505000	1.013139000	8.297344000

(o) $[Re(tacn)O_3]^+$ + dimethylketene transition state

C	0.643665000	2.296344000	5.969782000
C	1.439923000	1.270946000	9.436947000
C	1.924160000	1.689374000	8.044265000
C	-0.289843000	1.084390000	5.953715000
C	-0.445877000	-0.264562000	8.800512000
C	-1.581412000	-0.037844000	7.801860000
C	-3.699884000	5.924945000	7.401468000
C	-3.740879000	4.432123000	7.594582000
C	-3.922792000	3.970311000	8.912582000
C	-4.233761000	3.577369000	6.458456000
H	0.149414000	3.167061000	5.533496000
H	0.269957000	0.160857000	6.112190000
H	0.423514000	-0.705214000	8.307669000
H	1.407033000	3.575156000	7.370013000
H	1.545260000	2.082748000	5.382087000
H	1.626027000	2.067690000	10.158620000
H	1.993317000	0.380519000	9.761241000
H	2.043341000	0.817583000	7.398189000
H	2.915492000	2.143729000	8.133303000
H	-0.339187000	1.014425000	10.413960000
H	-0.759170000	0.994690000	4.969134000
H	-0.767131000	-0.982295000	9.561015000
H	-1.686844000	-0.912720000	7.148148000
H	-2.205767000	1.484705000	6.588276000
H	-2.525418000	0.108845000	8.330778000
H	-3.180719000	6.169765000	6.470087000
H	-3.192811000	6.434017000	8.223254000
H	-3.604116000	3.707694000	5.570540000
H	-4.287490000	2.516712000	6.717401000
H	-4.722874000	6.322426000	7.337081000
H	-5.251577000	3.883707000	6.179610000
N	0.977387000	2.651969000	7.375781000
N	-0.034058000	1.024371000	9.442342000
N	-1.330615000	1.203673000	7.022627000
O	-0.139290000	3.863318000	9.741802000
O	-1.643577000	4.092639000	7.367283000
O	-2.658492000	2.704366000	9.185413000
O	-4.542646000	4.091702000	9.909330000
Re	-0.981821000	2.968667000	8.561171000

(p) $[Re(tacn)O_3]^+$ + 2-butyne transition state

C	0.042013000	2.081040000	6.041788000
C	0.322953000	0.580841000	5.968688000
C	0.511630000	1.829679000	9.703470000
C	0.602297000	-0.538285000	8.872950000
C	0.867646000	2.502922000	8.377432000
C	-0.074514000	-1.212812000	7.677992000
C	-3.939548000	3.598997000	8.498832000
C	-4.137884000	3.312576000	7.305431000

C	-4.216020000	4.348421000	9.727848000
C	-4.826121000	3.477131000	6.021406000
H	0.304566000	0.252986000	4.929972000
H	0.651899000	-1.831767000	7.146428000
H	0.801724000	-1.283136000	9.642351000
H	0.900649000	2.642235000	5.666167000
H	1.175301000	3.532372000	8.558658000
H	1.317866000	0.355930000	6.349710000
H	1.416936000	1.638939000	10.283723000
H	1.565709000	-0.122418000	8.584142000
H	1.712319000	2.003303000	7.906321000
H	-0.148647000	2.467561000	10.287495000
H	-0.656957000	0.259676000	10.307029000
H	-0.758348000	3.359015000	7.430310000
H	-0.827211000	2.331161000	5.437370000
H	-0.889796000	-1.848965000	8.014662000
H	-1.316812000	-0.661513000	6.144626000
H	-3.318397000	4.845741000	10.098695000
H	-4.119859000	3.706186000	5.221742000
H	-4.573697000	3.681362000	10.513455000
H	-4.979348000	5.106439000	9.543029000
H	-5.352505000	2.562159000	5.745768000
H	-5.552174000	4.289688000	6.084128000
N	-0.236732000	0.571373000	9.436413000
N	-0.289656000	2.457943000	7.438635000
N	-0.674594000	-0.189418000	6.773894000
O	-2.548850000	2.376684000	9.226329000
O	-2.847750000	-0.359648000	8.589901000
O	-2.950715000	1.815335000	6.791813000
Re	-2.059090000	1.077809000	8.130827000

(q) $[Re(tacn)O_3]^+$ + norbornene transition state

C	0.097725000	1.901964000	5.986076000
C	0.301680000	0.388672000	6.037256000
C	0.567213000	-0.493399000	9.030093000
C	0.611608000	1.938021000	9.651381000
C	0.985130000	2.469739000	8.267703000
C	-0.154825000	-1.229544000	7.899021000
C	-3.799906000	3.847158000	8.418503000
C	-4.040083000	3.429965000	7.095097000
C	-5.072218000	3.570914000	9.191034000
C	-5.462238000	2.908747000	7.062889000
C	-5.595162000	2.306830000	8.477178000
C	-6.095334000	4.635319000	8.672914000
C	-6.363428000	4.178278000	7.205698000
H	0.249987000	-0.023218000	5.030254000
H	0.536460000	-1.923503000	7.415089000
H	0.732673000	-1.181930000	9.857889000
H	0.976673000	2.384067000	5.551995000
H	1.290521000	0.146064000	6.423466000
H	1.356567000	3.490690000	8.352980000

H	1.513557000	1.752318000	10.238795000
H	1.549647000	-0.157426000	8.703612000
H	1.790667000	1.880507000	7.833106000
H	-0.007231000	2.659545000	10.181121000
H	-0.600072000	3.336134000	7.270994000
H	-0.627622000	0.491653000	10.402654000
H	-0.769297000	2.143370000	5.374956000
H	-0.994101000	-1.796993000	8.294015000
H	-1.393584000	-0.749787000	6.338746000
H	-3.171212000	4.690744000	8.662508000
H	-3.609932000	3.928582000	6.238697000
H	-4.965541000	3.534539000	10.271793000
H	-4.991282000	1.418621000	8.642643000
H	-5.703979000	5.650282000	8.736843000
H	-5.707002000	2.272218000	6.217006000
H	-6.117558000	4.944410000	6.470841000
H	-6.628632000	2.079817000	8.737551000
H	-7.003038000	4.595327000	9.274951000
H	-7.408292000	3.905625000	7.057879000
N	-0.188768000	2.411966000	7.350003000
N	-0.204697000	0.703571000	9.503958000
N	-0.719010000	-0.258764000	6.917439000
O	-2.432464000	2.597199000	9.173561000
O	-2.849042000	-0.175393000	8.781013000
O	-2.900437000	1.828717000	6.776519000
Re	-2.017909000	1.184570000	8.179024000

(r)[Re(tacn)O_3]$^+$ + propene transition state

C	0.422333000	2.209980000	5.917057000
C	1.495726000	1.430729000	9.363267000
C	1.860750000	1.782705000	7.922445000
C	-0.317629000	-0.239255000	8.921397000
C	-0.434055000	0.950221000	6.017860000
C	-1.525922000	-0.141752000	7.994547000
C	-3.618132000	5.830404000	7.796997000
C	-3.754090000	4.352787000	7.561769000
C	-4.182819000	3.475970000	8.570096000
H	0.189107000	0.071631000	6.186371000
H	0.545299000	-0.641964000	8.389873000
H	1.198254000	3.595007000	7.197097000
H	1.301819000	2.017915000	5.292645000
H	1.683921000	2.276338000	10.025294000
H	1.972553000	0.879678000	7.322148000
H	2.112485000	0.590661000	9.702370000
H	2.826778000	2.292993000	7.906746000
H	-0.143525000	3.027274000	5.467621000
H	-0.197435000	1.118067000	10.461062000
H	-0.535907000	-0.929810000	9.739221000
H	-0.962536000	0.786873000	5.075442000
H	-1.605519000	-1.051047000	7.388130000
H	-2.328729000	1.259080000	6.748695000

H	-2.444842000	-0.037661000	8.572954000
H	-2.936276000	6.289711000	7.075224000
H	-3.263411000	6.049530000	8.808752000
H	-3.961813000	4.057809000	6.535624000
H	-4.422209000	3.874447000	9.551893000
H	-4.596169000	6.314484000	7.674000000
H	-4.706767000	2.567701000	8.291927000
N	0.045116000	1.104972000	9.473361000
N	0.819445000	2.654379000	7.279098000
N	-1.411603000	1.067522000	7.141946000
O	-0.251686000	3.947188000	9.648908000
O	-1.821098000	3.925387000	7.313774000
O	-2.635202000	2.497773000	9.271127000
Re	-1.073007000	2.927992000	8.560330000

(s) $[Tc(tacn)O_3]^+$ + dimethylketene transition state

C	0.459642000	1.816908000	5.786069000
C	1.538512000	1.676771000	9.327122000
C	1.902219000	1.766217000	7.841784000
C	-0.264727000	-0.066965000	9.201935000
C	-0.391853000	0.584905000	6.107639000
C	-1.472983000	-0.140531000	8.265521000
C	-3.662691000	5.484390000	8.449273000
C	-3.925649000	4.397139000	7.448215000
C	-4.172744000	3.087083000	7.834420000
C	-4.341250000	4.786030000	6.051893000
H	0.237891000	-0.251215000	6.417550000
H	0.601253000	-0.563805000	8.758477000
H	1.222048000	3.418501000	6.802802000
H	1.331601000	1.523352000	5.187635000
H	1.710570000	2.636898000	9.817015000
H	2.024444000	0.768588000	7.415650000
H	2.183440000	0.931203000	9.810895000
H	2.871723000	2.263972000	7.739855000
H	-0.120267000	2.540568000	5.208938000
H	-0.150596000	1.554277000	10.472347000
H	-0.490846000	-0.608809000	10.125478000
H	-0.914370000	0.258552000	5.202734000
H	-1.560628000	-1.152531000	7.850239000
H	-2.291022000	1.003888000	6.791736000
H	-2.390205000	0.081298000	8.813870000
H	-2.854236000	6.139074000	8.105636000
H	-3.409817000	5.103624000	9.437964000
H	-3.646762000	5.528381000	5.646980000
H	-4.383325000	3.936006000	5.369608000
H	-4.561259000	6.109015000	8.542364000
H	-5.339715000	5.244082000	6.086984000
N	0.096199000	1.350936000	9.506133000
N	0.861868000	2.497544000	7.044632000
N	-1.365483000	0.884151000	7.195897000
O	-0.256740000	4.169216000	9.160071000

O	-1.803431000	3.783580000	6.883575000
O	-2.669670000	2.662623000	8.946740000
O	-4.823839000	2.105586000	7.751590000
Tc	-1.063976000	2.992505000	8.252476000

(t) $[Tc(tacn)O_3]^+$ + 2-butyne transition state

C	0.039351000	2.105821000	6.058894000
C	0.317038000	0.605137000	5.964520000
C	0.515246000	1.801286000	9.712730000
C	0.596487000	-0.553582000	8.851874000
C	0.866785000	2.494294000	8.395014000
C	-0.085380000	-1.208551000	7.648015000
C	-4.008726000	3.660973000	8.494933000
C	-4.198736000	3.387351000	7.306260000
C	-4.218272000	4.345016000	9.771483000
C	-4.816339000	3.477343000	5.982476000
H	0.299363000	0.294969000	4.919951000
H	0.638346000	-1.827784000	7.112054000
H	0.796770000	-1.313176000	9.606985000
H	0.899713000	2.667967000	5.687165000
H	1.178453000	3.519783000	8.593037000
H	1.312857000	0.374950000	6.340389000
H	1.424399000	1.607802000	10.287032000
H	1.561277000	-0.138830000	8.565359000
H	1.710198000	2.000267000	7.915542000
H	-0.141487000	2.432439000	10.308245000
H	-0.655940000	0.226879000	10.300036000
H	-0.758444000	3.367624000	7.463590000
H	-0.828184000	2.366014000	5.456187000
H	-0.903139000	-1.845104000	7.978099000
H	-1.328130000	-0.631412000	6.124360000
H	-3.301438000	4.817369000	10.125852000
H	-4.073482000	3.678037000	5.209540000
H	-4.552734000	3.644615000	10.537457000
H	-4.980843000	5.117603000	9.652862000
H	-5.323458000	2.546671000	5.725638000
H	-5.550329000	4.285841000	5.972964000
N	-0.231462000	0.548505000	9.435771000
N	-0.289127000	2.467743000	7.457496000
N	-0.677978000	-0.175446000	6.756609000
O	-2.518974000	2.348795000	9.241747000
O	-2.822441000	-0.354206000	8.587404000
O	-2.922410000	1.789723000	6.795218000
Tc	-2.070546000	1.084819000	8.136641000

(u) $[Tc(tacn)O_3]^+$ + norbornene transition state

C	0.119254000	1.870829000	5.972894000
C	0.342720000	0.359747000	6.034912000
C	0.607122000	-0.495482000	9.023615000
C	0.610297000	1.934009000	9.637309000
C	0.977896000	2.473970000	8.253967000
C	-0.109806000	-1.250023000	7.902181000
C	-3.847270000	3.909880000	8.377469000
C	-4.119282000	3.486229000	7.083466000
C	-5.084743000	3.630807000	9.199060000
C	-5.528875000	2.939552000	7.090391000
C	-5.616367000	2.353597000	8.515464000
C	-6.136442000	4.680542000	8.699038000
C	-6.444662000	4.202345000	7.248160000
H	0.306817000	-0.055544000	5.028280000
H	0.584966000	-1.951232000	7.432828000
H	0.795317000	-1.177417000	9.852576000
H	0.995655000	2.359007000	5.539247000
H	1.325557000	3.503211000	8.344106000
H	1.332848000	0.134637000	6.428666000
H	1.516275000	1.770429000	10.226165000
H	1.580254000	-0.144085000	8.685312000
H	1.801639000	1.905029000	7.826141000
H	-0.021020000	2.645698000	10.165559000
H	-0.612283000	3.306049000	7.236029000
H	-0.612316000	0.471163000	10.385891000
H	-0.744322000	2.096268000	5.350688000
H	-0.947586000	-1.814716000	8.304706000
H	-1.342592000	-0.795721000	6.329763000
H	-3.141083000	4.685845000	8.630670000
H	-3.654116000	3.891548000	6.198174000
H	-4.943525000	3.609681000	10.275886000
H	-4.993309000	1.476982000	8.672292000
H	-5.752591000	5.699325000	8.738292000
H	-5.791069000	2.293801000	6.257423000
H	-6.235052000	4.961982000	6.496116000
H	-6.639147000	2.117776000	8.808264000
H	-7.023326000	4.638637000	9.331034000
H	-7.488538000	3.909147000	7.137993000
N	-0.179716000	0.686600000	9.493171000
N	-0.184158000	2.391312000	7.326258000
N	-0.672855000	-0.297823000	6.907478000
O	-2.387743000	2.520071000	9.162022000
O	-2.803892000	-0.214985000	8.747104000
O	-2.857286000	1.745262000	6.748577000
Tc	-2.015433000	1.152250000	8.152108000

(v) $[Tc(tacn)O_3]^+$ + propene transition state

C	0.446606000	2.191660000	5.902826000
C	1.483658000	1.443914000	9.375810000
C	1.868099000	1.797415000	7.934738000
C	-0.309765000	-0.255367000	8.926010000
C	-0.405322000	0.922267000	5.997732000
C	-1.507741000	-0.171854000	7.976563000
C	-3.612214000	5.858463000	7.824345000
C	-3.808166000	4.391858000	7.584912000
C	-4.211740000	3.510512000	8.599025000
H	0.223964000	0.046555000	6.168657000
H	0.562921000	-0.664560000	8.412209000
H	1.187803000	3.598934000	7.186353000
H	1.325445000	2.003602000	5.272565000
H	1.642709000	2.302348000	10.030490000
H	2.010776000	0.892094000	7.341471000
H	2.124438000	0.625517000	9.729862000
H	2.832978000	2.314215000	7.940989000
H	-0.128572000	3.002207000	5.450159000
H	-0.221990000	1.122212000	10.457438000
H	-0.541176000	-0.950728000	9.738743000
H	-0.913543000	0.753303000	5.042901000
H	-1.580337000	-1.096295000	7.388825000
H	-2.312184000	1.219306000	6.715388000
H	-2.431862000	-0.060438000	8.547348000
H	-2.946625000	6.301020000	7.078521000
H	-3.214345000	6.061503000	8.822633000
H	-4.043024000	4.108300000	6.560836000
H	-4.399672000	3.898074000	9.596047000
H	-4.579665000	6.371673000	7.740367000
H	-4.771483000	2.618915000	8.336588000
N	0.040641000	1.089369000	9.474864000
N	0.831770000	2.648573000	7.262587000
N	-1.395548000	1.026676000	7.109435000
O	-0.283170000	3.939801000	9.602888000
O	-1.850728000	3.903812000	7.273182000
O	-2.662158000	2.492789000	9.235167000
Tc	-1.107570000	2.928073000	8.528594000

E. Selected PBE-D2 optimized coordinates (Å)

(a) $[Re(tacn)O_3]^+$ + dimethylketene transition state

C	2.662041000	1.946266000	0.625058000
C	2.905586000	-0.569349000	0.680011000
C	3.105551000	0.642217000	0.040631000
C	4.061388000	0.665339000	-1.120129000
C	-1.121788000	1.354322000	2.037119000
C	-1.556442000	2.356227000	-0.226167000
C	-1.613228000	1.726414000	-1.614459000
C	-2.264779000	0.345693000	2.003599000
C	-2.981240000	-1.210927000	0.176390000
C	-3.114606000	-0.160936000	-0.924958000
H	0.264241000	2.057253000	0.691764000
H	2.060019000	1.802302000	1.533820000
H	2.107193000	2.543168000	-0.123087000
H	3.545924000	2.544452000	0.911113000
H	3.640164000	1.274385000	-1.937001000
H	4.269658000	-0.342249000	-1.505719000
H	5.018640000	1.127304000	-0.812825000
H	-0.271961000	0.953082000	2.607132000
H	-0.666302000	1.887255000	-2.148100000
H	-1.209016000	3.395802000	-0.292507000
H	-1.451211000	2.303656000	2.486525000
H	-1.637782000	-0.179696000	-2.382241000
H	-1.688371000	-1.620632000	1.722569000
H	-2.445041000	2.144774000	-2.200934000
H	-2.493955000	-0.007182000	3.017618000
H	-2.549111000	2.371736000	0.243260000
H	-2.684651000	-2.178561000	-0.248079000
H	-3.177722000	0.804974000	1.600599000
H	-3.617365000	0.738151000	-0.545153000
H	-3.728691000	-0.555424000	-1.744933000
H	-3.939051000	-1.323395000	0.707822000
N	-0.632152000	1.561240000	0.645237000
N	-1.762321000	0.248332000	-1.458235000
N	-1.893033000	-0.812302000	1.125569000
O	1.123351000	0.307290000	-1.226222000
O	1.184738000	-0.574971000	1.129974000
O	3.372260000	-1.632293000	0.956378000
O	-0.286568000	-2.092745000	-0.767229000
Re	-0.039709000	-0.498025000	-0.194339000

(b) $[Re(tacn)O_3]^+$ + 2-butyne transition state

C	3.004590000	0.634398000	0.238683000
C	3.006961000	-0.626893000	0.263081000
C	3.660541000	1.937291000	0.397929000
C	3.668398000	-1.919771000	0.471065000

C	-0.816187000	-1.400038000	2.056327000
C	-1.228545000	1.071882000	2.188114000
C	-1.398742000	2.119731000	1.089648000
C	-2.029276000	-1.741020000	1.195425000
C	-2.931355000	-0.627317000	-0.859685000
C	-3.027881000	0.776527000	-0.267099000
H	0.017663000	-2.077876000	1.830008000
H	0.599181000	0.097340000	2.063767000
H	3.118676000	2.556999000	1.131404000
H	3.134750000	-2.509408000	1.234787000
H	3.667437000	2.490791000	-0.555514000
H	3.668625000	-2.512893000	-0.458307000
H	4.701191000	1.811607000	0.739234000
H	4.711623000	-1.776967000	0.797575000
H	-0.465040000	2.680239000	0.946703000
H	-0.798785000	1.531266000	3.088486000
H	-1.070068000	-1.470296000	3.125658000
H	-1.603964000	2.116711000	-0.953966000
H	-1.612629000	-2.204161000	-0.777673000
H	-2.198941000	0.641998000	2.472004000
H	-2.216289000	2.814552000	1.335713000
H	-2.253296000	-2.814045000	1.256415000
H	-2.719579000	-0.576892000	-1.936013000
H	-2.918569000	-1.200258000	1.547230000
H	-3.439005000	0.742209000	0.750454000
H	-3.703744000	1.395583000	-0.871583000
H	-3.872018000	-1.176210000	-0.696007000
N	-0.350290000	-0.033478000	1.697912000
N	-1.666758000	1.420074000	-0.203845000
N	-1.778415000	-1.350441000	-0.234946000
O	1.223493000	-1.278715000	-0.169781000
O	1.226472000	1.257617000	-0.239759000
O	-0.260386000	-0.070345000	-2.275378000
Re	0.038045000	-0.019074000	-0.587277000

(c) $[Re(tacn)O_3]^+$ + norbornene transition state

C	1.773484000	1.915933000	-1.479794000
C	2.147329000	0.976628000	2.077017000
C	2.231236000	2.040356000	0.984591000
C	2.755616000	0.789585000	-1.785888000
C	3.172011000	-1.370774000	-0.593182000
C	3.409715000	-0.767746000	0.790038000
C	-2.438985000	0.904455000	-0.727991000
C	-2.439265000	0.926074000	0.695675000
C	-3.245106000	-1.193088000	0.015773000
C	-3.426253000	-0.176018000	-1.133726000
C	-3.426740000	-0.141550000	1.133767000
C	-4.838232000	0.401566000	-0.791035000
C	-4.838454000	0.425582000	0.773031000
H	0.424626000	2.323536000	0.017032000
H	0.903787000	1.858002000	-2.147915000

H	1.207694000	1.077384000	2.637069000
H	1.878186000	-1.057687000	2.156717000
H	1.894770000	-1.049662000	-2.174487000
H	2.030962000	3.034909000	1.405376000
H	2.262677000	2.896284000	-1.591767000
H	2.722915000	-2.368908000	-0.505591000
H	2.980164000	0.758411000	-2.860261000
H	3.002621000	1.056159000	2.765396000
H	3.236408000	2.069062000	0.542559000
H	3.705838000	0.948848000	-1.257650000
H	3.932759000	-1.490277000	1.430176000
H	4.044387000	0.125580000	0.720430000
H	4.122687000	-1.443035000	-1.144382000
H	-2.262183000	-1.682629000	0.023338000
H	-2.324189000	1.851026000	1.263647000
H	-2.324434000	1.812050000	-1.323260000
H	-3.320513000	-0.548131000	-2.161364000
H	-3.321509000	-0.482193000	2.172307000
H	-4.036832000	-1.958898000	0.027330000
H	-4.992531000	1.397601000	-1.233389000
H	-4.992584000	1.434783000	1.184540000
H	-5.620896000	-0.269316000	-1.178704000
H	-5.621374000	-0.232858000	1.180955000
N	1.249539000	1.727233000	-0.099468000
N	2.105047000	-0.369661000	1.431017000
N	2.186572000	-0.528181000	-1.341848000
O	0.288484000	-2.122227000	0.022363000
O	-0.730066000	0.192222000	-1.288825000
O	-0.735130000	0.224564000	1.275979000
Re	0.347654000	-0.408110000	0.003976000

(d)$[Re(tacn)O_3]^+$ + propene transition state

C	1.296645000	0.236072000	2.369239000
C	1.337838000	2.234132000	-0.741959000
C	1.515643000	1.578635000	1.675259000
C	2.340084000	1.214941000	-1.274041000
C	2.601779000	-1.222336000	-0.770972000
C	2.683156000	-1.084147000	0.748056000
C	-2.869707000	1.192803000	-0.805410000
C	-3.116340000	0.689141000	0.504774000
C	-4.004966000	-0.512518000	0.686012000
H	0.299872000	0.201995000	2.829824000
H	0.547858000	2.417408000	-1.482767000
H	1.004759000	-1.711448000	1.790647000
H	1.276638000	2.403584000	2.359706000
H	1.517318000	-0.378094000	-2.302520000
H	1.843263000	3.181133000	-0.495996000
H	2.068084000	0.061706000	3.134815000
H	2.139726000	-2.179716000	-1.045576000
H	2.565291000	1.698728000	1.374336000
H	2.687503000	1.504509000	-2.274515000

H	3.121998000	-1.990556000	1.185351000
H	3.223892000	1.163971000	-0.623202000
H	3.328914000	-0.241140000	1.027126000
H	3.609015000	-1.161481000	-1.212146000
H	-0.165989000	2.221661000	0.660321000
H	-2.699639000	2.265174000	-0.923954000
H	-3.124583000	1.419689000	1.318395000
H	-3.392853000	0.714196000	-1.638052000
H	-3.806627000	-1.256889000	-0.102180000
H	-3.837990000	-0.983246000	1.666662000
H	-5.068425000	-0.222457000	0.622208000
N	0.661193000	1.655210000	0.450368000
N	1.315579000	-0.845411000	1.338126000
N	1.713648000	-0.150071000	-1.322950000
O	-0.339280000	-2.006341000	-0.719886000
O	-1.198224000	0.647447000	-1.352683000
O	-1.494279000	-0.091280000	1.063973000
Re	-0.258953000	-0.374744000	-0.205390000

(e) $[Tc(tacn)O_3]^+$ + dimethylketene transition state

C	1.204640000	2.082380000	-1.071158000
C	1.496085000	1.813790000	1.403765000
C	1.513034000	0.562250000	2.280998000
C	2.342972000	1.160114000	-1.499662000
C	2.977704000	-1.113976000	-0.675475000
C	3.061681000	-0.748168000	0.805794000
C	-2.603415000	1.004943000	-0.542840000
C	-3.245491000	-0.055193000	0.039433000
C	-3.391577000	-1.385467000	-0.634393000
C	-3.975811000	0.201791000	1.333212000
H	0.386233000	2.053745000	-1.803045000
H	0.546096000	0.436413000	2.787436000
H	1.129442000	2.672891000	1.982493000
H	1.507787000	-1.471729000	1.975429000
H	1.569787000	3.117401000	-0.971200000
H	1.745868000	-0.668844000	-2.265340000
H	2.315306000	0.629358000	3.032802000
H	2.509405000	2.062449000	1.059437000
H	2.620742000	1.361808000	-2.543221000
H	2.659580000	-2.158177000	-0.795852000
H	3.237033000	1.338254000	-0.885796000
H	3.576901000	0.212626000	0.939237000
H	3.647167000	-1.506141000	1.344244000
H	3.962103000	-0.976738000	-1.151830000
H	-0.276347000	2.038416000	0.344707000
H	-2.789272000	-2.148720000	-0.108547000
H	-3.062661000	-1.357073000	-1.681094000
H	-3.706915000	1.167847000	1.781473000
H	-3.741767000	-0.597837000	2.054069000
H	-4.446476000	-1.703315000	-0.586008000
H	-5.065074000	0.188607000	1.146647000

N	0.635348000	1.589289000	0.207471000
N	1.693655000	-0.634558000	1.414344000
N	1.941807000	-0.271842000	-1.341512000
O	0.215814000	-2.302565000	-0.415926000
O	-1.057436000	0.090009000	-1.349973000
O	-1.159448000	-0.396610000	1.142929000
O	-2.425965000	2.160642000	-0.740094000
Tc	0.021320000	-0.628327000	-0.124458000

(f) $[Tc(tacn)O_3]^+$ + 2-butyne transition state

C	3.115374000	0.624510000	0.144727000
C	3.118968000	-0.623331000	0.158789000
C	3.634307000	1.986912000	0.285030000
C	3.646089000	-1.979166000	0.326555000
C	-0.755606000	-1.407690000	1.940497000
C	-1.168280000	1.058434000	2.090325000
C	-1.376950000	2.109753000	1.000139000
C	-1.987835000	-1.751644000	1.105499000
C	-2.938125000	-0.635489000	-0.923043000
C	-3.027751000	0.766256000	-0.322260000
H	0.073961000	-2.085451000	1.698225000
H	0.661617000	0.090910000	1.906773000
H	3.043688000	2.557025000	1.020078000
H	3.066704000	-2.533626000	1.082258000
H	3.580879000	2.529619000	-0.672332000
H	3.584562000	-2.545626000	-0.616478000
H	4.685436000	1.962542000	0.617684000
H	4.700845000	-1.941660000	0.646408000
H	-0.453349000	2.681483000	0.836492000
H	-0.719382000	1.521475000	2.980279000
H	-0.989265000	-1.486122000	3.014728000
H	-1.597541000	-2.198545000	-0.879610000
H	-1.617091000	2.104818000	-1.042896000
H	-2.129850000	0.625721000	2.399916000
H	-2.190538000	2.798208000	1.279587000
H	-2.204288000	-2.826553000	1.172961000
H	-2.748456000	-0.579459000	-2.003281000
H	-2.871175000	-1.218383000	1.484000000
H	-3.417263000	0.722484000	0.703908000
H	-3.725934000	1.380990000	-0.906800000
H	-3.877927000	-1.183221000	-0.745507000
N	-0.301700000	-0.042056000	1.583279000
N	-1.678552000	1.419764000	-0.283592000
N	-1.779488000	-1.357875000	-0.323724000
O	1.175066000	-1.301581000	-0.336937000
O	1.177531000	1.277076000	-0.387067000
O	-0.340328000	-0.055105000	-2.386209000
Tc	0.041936000	-0.017904000	-0.715442000

(g) $[Tc(tacn)O_3]^+$ + norbornene transition state

C	1.856362000	1.823393000	-1.497318000
C	2.255700000	0.923509000	2.063233000
C	2.313653000	1.978882000	0.959504000
C	2.846140000	0.698113000	-1.792671000
C	3.275766000	-1.447714000	-0.581116000
C	3.518403000	-0.826861000	0.794019000
C	-2.564463000	0.962085000	0.659521000
C	-2.565882000	0.920900000	-0.720554000
C	-3.241633000	-1.211276000	0.034357000
C	-3.475034000	-0.153015000	1.137059000
C	-3.477274000	-0.220601000	-1.128969000
C	-4.927144000	0.318949000	0.772025000
C	-4.928798000	0.272105000	-0.789874000
H	0.495911000	2.230232000	-0.005783000
H	0.989829000	1.759599000	-2.169135000
H	1.325604000	1.021105000	2.639549000
H	1.972082000	-1.145747000	-2.147298000
H	1.977121000	-1.113736000	2.152271000
H	2.105617000	2.974089000	1.376374000
H	2.344744000	2.803404000	-1.621815000
H	2.827375000	-2.444996000	-0.479474000
H	3.067069000	0.660493000	-2.868249000
H	3.119505000	1.027732000	2.739067000
H	3.316854000	2.016982000	0.513020000
H	3.797803000	0.874165000	-1.271875000
H	4.051823000	-1.539557000	1.437853000
H	4.151022000	0.067097000	0.707537000
H	4.227396000	-1.529878000	-1.130838000
H	-2.228795000	-1.636179000	0.046035000
H	-2.286858000	1.823197000	1.266966000
H	-2.289156000	1.744267000	-1.378504000
H	-3.346071000	-0.468675000	2.180170000
H	-3.350421000	-0.597928000	-2.151660000
H	-3.987919000	-2.021653000	0.059382000
H	-5.145098000	1.320997000	1.170822000
H	-5.147930000	1.248399000	-1.247553000
H	-5.662746000	-0.384817000	1.192090000
H	-5.665009000	-0.455793000	-1.165437000
N	1.334834000	1.654070000	-0.118260000
N	2.225938000	-0.424539000	1.436390000
N	2.296572000	-0.614938000	-1.333877000
O	0.434519000	-2.195551000	0.058337000
O	-0.554651000	0.050802000	-1.326560000
O	-0.558625000	0.126723000	1.312339000
Tc	0.429166000	-0.479571000	0.010663000

(h)[Tc(tacn)O_3]$^+$ + propene transition state

C	1.226172000	-0.305333000	2.325489000
C	1.253011000	2.291391000	-0.303457000
C	1.412224000	1.157701000	1.925664000
C	2.285661000	1.424337000	-1.019776000
C	2.592616000	-1.057677000	-1.026969000
C	2.656931000	-1.233618000	0.489740000
C	-3.035332000	1.260113000	-0.603601000
C	-3.284587000	0.499652000	0.540754000
C	-4.098257000	-0.762357000	0.484877000
H	0.228242000	-0.460021000	2.758365000
H	0.471322000	2.614220000	-1.004517000
H	0.973448000	-2.093873000	1.342511000
H	1.145940000	1.815186000	2.764960000
H	1.495169000	0.060602000	-2.363173000
H	1.738271000	3.177261000	0.137854000
H	1.994958000	-0.606104000	3.055039000
H	2.150141000	-1.945662000	-1.497587000
H	2.462398000	1.361967000	1.675218000
H	2.637324000	1.928115000	-1.930623000
H	3.117448000	-2.200906000	0.733544000
H	3.164123000	1.261961000	-0.379254000
H	3.284872000	-0.454365000	0.942663000
H	3.605846000	-0.898709000	-1.430487000
H	-0.278029000	1.951725000	1.031162000
H	-2.786049000	2.318342000	-0.512390000
H	-3.216183000	1.000081000	1.510263000
H	-3.456987000	0.939524000	-1.559351000
H	-3.817827000	-1.453277000	1.294100000
H	-3.964072000	-1.272010000	-0.482356000
H	-5.171484000	-0.527241000	0.600841000
N	0.575808000	1.472846000	0.732300000
N	1.289374000	-1.149695000	1.102069000
N	1.700317000	0.090009000	-1.360316000
O	-0.298317000	-1.889311000	-1.176816000
O	-1.184216000	0.809816000	-1.290766000
O	-1.494380000	-0.433758000	0.955007000
Tc	-0.301690000	-0.394143000	-0.343742000
